# Anti-*Leishmania* activated C-kinase monoclonal antibody immunohistochemical technique for cutaneous leishmaniasis diagnosis

**DOI:** 10.1371/journal.pone.0339746

**Published:** 2026-08-05

**Authors:** Amanda Desirée Lopes Santos, Marcelo Antônio Pascoal-Xavier, Vanessa Peruhype-Magalhães, Marcela Tavares Caldas Eller, Francislane Ramalho Varella Pereira, Maryana Prates Rodrigues, Maria Nilza Pereira de Brito, Bruno Oliveira Souza e Silva, Thadeu Ramalho da Silva, Daniel Moreira de Avelar, Mariana Lourenço Freire, Edward Oliveira

**Affiliations:** 1 Grupo de Genômica Funcional de Parasitos, Instituto René Rachou, Fundação Oswaldo Cruz, Fiocruz, Belo Horizonte, Minas Gerais, Brazil; 2 Grupo de Imunologia das Doenças Virais, Instituto René Rachou, Fundação Oswaldo Cruz, Fiocruz, Belo Horizonte, Minas Gerais, Brazil; 3 Departamento de Anatomia Patológica e Medicina Legal, Faculdade de Medicina, Universidade Federal de Minas Gerais, Belo Horizonte, Minas Gerais, Brazil; 4 Grupo Integrado de Pesquisas em Biomarcadores, Instituto René Rachou, Fundação Oswaldo Cruz, Fiocruz, Belo Horizonte, Minas Gerais, Brazil; 5 Serviço de Atendimento Especializado Ampliado -SAE-A, Teófilo Otoni, Minas Gerais, Brazil; 6 Secretaria Municipal de Saúde, Teófilo Otoni, Minas Gerais, Brazil; 7 Superintendência Regional de Saúde-SRS, Teófilo Otoni, Minas Gerais, Brazil; 8 Laboratório Macrorregional de Teófilo Otoni, Teófilo Otoni, Minas Gerais, Brazil; 9 Grupo de Pesquisa Clínica e Políticas Públicas em Doenças Infecto-Parasitárias, Instituto René Rachou, Fundação Oswaldo Cruz, Fiocruz, Belo Horizonte, Minas Gerais, Brazil; Indian Institute of Technology (IIT BHU) - Varanasi, INDIA

## Abstract

This study aimed to develop and validate an immunohistochemistry (IHC) technique using a monoclonal antibody (mAb) against the *Leishmania* homolog of activated C kinase (LACK) antigen (anti-LACK IHC) for the diagnosis of cutaneous leishmaniasis (CL). The *Leishmania braziliensis* LACK sequence (A4HGX7_LEIBR) was analyzed for B-cell epitope prediction, and three antigenic regions were selected to design a multiepitope antigen. The corresponding gene was back-translated, synthesized, and cloned into the pET28a(+) plasmid. Recombinant LACK protein was expressed and used as an immunogen for mAb production by somatic hybridization. The ability of the anti-LACK mAb to recognize native LACK and amastigotes of *Leishmania (Leishmania) amazonensis*, *Leishmania (Viannia) braziliensis*, and *Leishmania (Viannia) guyanensis* was confirmed by Western blotting using total parasite extracts and by IHC in skin histological sections from experimentally infected hamsters. Subsequently, the anti-LACK IHC assay was validated using skin lesion samples from 104 patients with suspected CL who attended the outpatient clinic of the Municipal Polyclinic of Teófilo Otoni, Brazil, between 2019 and 2020, using kDNA-PCR as the reference standard. The diagnostic performance of anti-LACK IHC was compared with direct examination (DE) and conventional histopathology (HE). Anti-LACK IHC showed a sensitivity of 59.3% (95% CI: 40.0–83.7), numerically higher than DE (53.7; *p* = 0.55) and HE (42.6; *p* = 0.08). The specificity of the anti-LACK IHC was 98.0% (95% CI: 89.5–99.7), equal to the DE (98.0%) and slightly higher to the HE (96.0%), but without statistically significant difference (*p* = 0.56). The accuracy was of 77.9% (95% CI: 61.9–96.8) with anti-LACK IHC, 75.0% with DE (*p* = 0.62) and 68.3% with HE (*p* = 0.12). The combination of anti-LACK IHC with DE or HE numerically increased diagnostic accuracy to 83.7% (*p* = 0.48) and 79.8% (*p* = 0.33), respectively. The results demonstrated that anti-LACK IHC was able to detect the main *Leishmania* species responsible for CL in Brazil and further studies may prove its applicability in the CL diagnosis routine in different Brazilian endemic regions.

## Introduction

Cutaneous leishmaniasis (CL) remains endemic in several regions worldwide, including the Americas, Africa, the Eastern Mediterranean, and Southeast Asia, with approximately 272,098 new cases reported globally in 2023 [1]. Although an overall 8% reduction in cases was observed in the Americas, the Pan American Health Organization (PAHO) still reported 34,954 cases in the region in 2023, of which 12,910 occurred in Brazil [2]. In Brazil, CL is mainly caused by *Leishmania (Viannia) braziliensis*, *Leishmania (Leishmania) amazonensis*, and *Leishmania (Viannia) guyanensis* [3]. Other species, including *L. (V.) naiffi*, *L. (V.) shawi*, *L. (V.) lindenbergi*, *L. (V.) lainsoni*, and *L. (L.) infantum*, have also been identified, particularly in the North and Northeast regions [4–8]. The high incidence, broad geographic distribution, and potential progression to chronic or mucosal forms make CL a persistent and significant public health challenge.

Accurate laboratory diagnosis is essential for appropriate clinical management of CL, especially considering the toxicity of available treatments and the clinical resemblance of CL lesions to other conditions, such as sporotrichosis, vascular ulcers, leprosy, paracoccidioidomycosis, skin cancer, and deep mycoses [9]. In Brazil, laboratory diagnosis is primarily based on direct examination (DE), which consists of microscopic evaluation of Giemsa-stained tissue imprints for rapid parasite detection, parasite culture, and, when available, polymerase chain reaction (PCR) and histopathological examination (HE) of formalin-fixed sained with hematoxylin–eosin [10]. However, these techniques often show limitations related to low sensitivity or technical complexity, which restricts their use in many endemic regions. Improving the performance of diagnostic tools is therefore a priority, in line with the United Nations 2030 Agenda, which aims to eliminate neglected tropical diseases as a public health problem by the end of the decade [11].

In different clinical settings in which a biopsy is performed as part of the standard diagnostic workup, but the diagnosis cannot be confirmed due to limitations of the available techniques, immunohistochemistry (IHC) emerges as a promising diagnostic alternative, allowing the detection of parasite antigens in tissue samples through the use of specific antibodies [12]. Despite methodological advances, the application of IHC for CL diagnosis remains limited. Most published studies rely on hyperimmune sera or biotin-based detection systems and report sensitivity values ranging from 58.5% to 91.8%, while data on specificity are scarce or absent [13–19]. The use of monoclonal antibodies (mAb) in IHC for CL diagnosis has been reported in only a few studies. Shirian et al. (2014) achieved sensitivity above 94% in *Old World* species [20], while Freire et al. (2022) [21] reported sensitivity up to 85.7% for *New World* species [21]. These findings highlight the potential of mAb-based IHC but also underscore the need for further optimization and validation.

The *Leishmania* activated C kinase (LACK) is a highly conserved protein among *Leishmania* species and is expressed in both promastigote and amastigote stages. LACK plays essential roles in parasite survival, temperature adaptation, and virulence, and is abundantly expressed during mammalian infection [22,23]. The protein is predominantly localized in the parasite cytoplasm, particularly near the kinetoplast, but has also been detected on the parasite surface, likely due to reassociation of its secreted form [24–26]. Because of its high expression, conservation across species, and critical role in parasite fitness [22,23], LACK has been extensively investigated as a molecular target. Previous studies demonstrated that genetic deletion of LACK significantly reduces parasite burden in vivo, reinforcing its relevance for parasite survival and virulence. Importantly, an immunohistochemical assay using an anti-LACK monoclonal antibody achieved 100% sensitivity for detecting amastigotes in visceral leishmaniasis tissue samples, highlighting its diagnostic potential [22]. Despite these promising findings, the performance of anti-LACK–based IHC has not yet been evaluated for the diagnosis of CL. Therefore, the present study aimed to develop and validate an IHC technique employing a mAb against LACK, combined with a polymer-based detection system, for the diagnosis of CL.

## Materials and methods

### Production of the recombinant LACK antigen

The amino acid sequences of the LACK protein from *L. braziliensis* (ID: A4HGX7_LEIBR), *L. amazonensis* (ID: Q95NJ3), and *L. guyanensis* (ID: A0A1E1J0L7) were compared using the Clustal Omega program. The *L. amazonensis* sequence showed ten amino acid differences at positions 43, 58, 99, 148, 183, 231, 237, 254, 256, and 277, resulting in 96.79% overall identity.

Subsequently, the *L. braziliensis* LACK sequence was analyzed using BCPRED and Bepipred 2.0 for B-cell epitope prediction. Three antigenic regions were selected (S1 File), reverse-translated, and designed a synthetic gene containing a start codon, *BamHI* and *HindIII* restriction sites, and a stop codon. Sequence design and optimization were performed using BioEdit v7.2.5 (Raleigh, NC, USA). The final 645-bp construct, including a 630-bp open reading frame encoding a 210-amino-acid protein (23.1 kDa), was cloned into the pET28a(+) vector for heterologous expression in *Escherichia coli* BL21 Star cells.

Following heat-shock transformation, recombinant clones were screened by *BamHI/HindIII* digestion and cultured in Luria–Bertani (LB) medium supplemented with kanamycin (30 µg/mL). Protein expression was performed as described by Freire et al. (2022) [21]: an 80-mL pre-culture was expanded to 2 L of LB medium, induced with 0.75 mM IPTG at OD₅₉₀ 0.6–0.8, and incubated overnight at 16 °C with shaking (200 rpm). Bacterial cells were harvested (10,000 × g, 20 min) and lysed under denaturing conditions to isolate inclusion bodies.

Cells were resuspended in lysis buffer (50 mM Tris-HCl, 500 mM NaCl, 0.2 mM EDTA, 3% sucrose, 1% Triton X-100, 200 µg/mL lysozyme, 1 mM PMSF, 20 µg/mL DNase I; pH 8.0) and sonicated on ice for five 30-s cycles (VC-750, Sonics Vibra-Cell, Sonics & Materials, Newton, CT, USA). Inclusion bodies were pelleted (13,000 × g, 40 min), washed with Tris–urea buffer (50 mM Tris-HCl, 3 M urea, 0.2 mM EDTA, 500 mM NaCl, pH 8.0), re-sonicated, and centrifuged again. Solubilization was achieved in phosphate buffer (10 mM) containing NaCl (200 mM), Tris-HCl (10 mM), guanidine-HCl (6 M), and β-mercaptoethanol (10 mM).

Recombinant LACK was purified using Ni Sepharose HP resin in Poly-Prep chromatography columns (GE Healthcare). Protein expression and purity were confirmed by SDS-PAGE. For Western blotting, proteins were transferred to nitrocellulose membranes and probed with anti-6 × His antibody (1:3,000; Thermo Fisher) followed by enhanced chemiluminescence (ECL) detection (GE Healthcare). Images were acquired using an ImageQuant LAS 4000 system.

### Production of the anti-LACK monoclonal antibody

Two five-week-old female BALB/c mice were subcutaneously immunized with 20 µg of recombinant LACK (rLACK) protein emulsified in Freund’s complete adjuvant, followed by four booster doses (20 µg each) in Freund’s incomplete adjuvant at 15-day intervals. Control mice received saline with adjuvant following the same immunization schedule. Blood samples were collected from the submandibular vein prior to each immunization.

Serum IgG antibody levels were determined by indirect ELISA using rLACK as the coating antigen. Microplates (Nunc Maxisorp™) were coated with 1 µg/mL rLACK in carbonate–bicarbonate buffer (pH 9.6) and incubated for 1 h at 37 °C, followed by overnight incubation at 2–8 °C. After washing with PBS containing 0.05% Tween-20 (PBS-T20), wells were blocked with 5% skim milk in PBS-T20 for 2 h at 37 °C. Serum samples (1:100 dilution in 1% PBS-T20-milk) were added and incubated for 1 h at 37 °C. Plates were washed and incubated with peroxidase-conjugated anti-mouse IgG (1:20,000; Thermo Fisher Scientific), followed by addition of TMB substrate. After 5 min, the reaction was stopped with 1 N H_2_SO_4_, and absorbance was measured at 450/620 nm using a Varioskan LUX reader (Thermo Fisher Scientific).

Following confirmation of specific IgG production, mice received an intraperitoneal booster dose of rLACK. Three days later, animals were euthanized by ketamine (500 mg/kg) and xylazine (50 mg/kg) anesthesia, and spleens were collected for somatic cell fusion with *Sp2/0-IL6* myeloma cells (3.5 × 10^7^ splenocytes: 7 × 10^6^ myeloma cells), as described by Freire et al. (2022) [21]. Fusion was performed using PEG-DMSO (Hybri-Max), and cells were washed and cultured in DMEM supplemented with 20% fetal bovine serum (FBS) and 1% Penicillin–Streptomycin for 24 h at 37 °C under 5% CO_2_. Subsequently, a selective HAT medium (2%) was added to 96-well plates previously seeded with murine peritoneal macrophages.

After 10–14 days, hybridoma supernatants were screened by ELISA. The highest-producing clone (absorbance ≥ 1.20) was subcloned by limiting dilution to obtain a monoclonal population. The selected clone was expanded, and culture supernatants containing the anti-LACK mAb were collected weekly. Supernatants were concentrated using the Amicon® Stirred Cell system with Diaflo ultrafilters and purified sequentially on HiTrap Blue HP and rProtein A FF affinity columns (GE Healthcare).

Purity of the anti-LACK mAb was assessed by 15% SDS-PAGE. Antigen recognition was confirmed by Western blot analysis using 1 µg of rLACK as antigen and anti-LACK mAb at a 1:100 dilution.

### Recognition of native LACK antigen in soluble *Leishmania* spp. antigens

The ability of the anti-LACK mAb to recognize the native antigen in total protein extracts from the main *Leishmania* species causing CL in Brazil was evaluated by Western blotting. Total extracts of *L. (L.) amazonensis*, *L. (V.) braziliensis*, and *L. (V.) guyanensis* (as described by Freire et al., 2022 [21]) were resolved by 15% SDS-PAGE.

Separated proteins were electrotransferred onto nitrocellulose membranes (Amersham Protran, 0.45 µm; GE Healthcare, UK) [27]. Membranes were blocked in 5% skim milk in PBS–Tween 20 (0.05%) and incubated with the anti-LACK mAb (1:3,000) for 1 h at room temperature. After washing, membranes were incubated with horseradish peroxidase (HRP)–conjugated anti-mouse IgG (Sigma-Aldrich, St. Louis, MO, USA) for 1 h. Immunoreactive bands were detected using ECL™ Prime Western Blotting Detection Reagent (GE Life Sciences, UK), and images were acquired using the ImageQuant LAS 4000 system (GE Healthcare, Chicago, IL, USA).

### Detection of amastigote forms of *Leishmania* spp. in histological sections from experimentally infected animals

To evaluate whether the anti-LACK mAb was able to detect amastigote forms of different *Leishmania* species in tissue sections, IHC was performed using skin lesions obtained from hamsters experimentally infected with the three main *Leishmania* species causing CL in Brazil. These experimentally infected animals were used exclusively as a source of tissue containing amastigotes for subsequent immunohistochemical evaluation.

Five-week-old male golden hamsters (*Mesocricetus auratus*) were inoculated subcutaneously in the right hind paw with 1 × 10^6^ promastigotes of either *L. (L.) amazonensis* (IFLA/BR/67/PH8), *L. (V.) braziliensis* (MHOM/BR/75/M2903), or *L. (V.) guyanensis* (MHOM/BR/75/M4147). Skin lesion fragments were collected 30–40 days after inoculation, fixed in 10% neutral buffered formalin (pH 7.2), processed for histology, and sectioned at 4 µm.

Tissue sections were subjected to IHC using the anti-LACK mAb following the protocol described by Freire et al. (2022) [21], with the antibody diluted 1:100. Parasite labeling was visualized using the Bond Polymer Refine Red Detection system (Leica Microsystems, Newcastle, UK), a biotin-free alkaline phosphatase polymer detection system that produces a red/burgundy signal.

### Standardization of the IHC protocol using anti-LACK monoclonal antibody

IHC using the anti-LACK mAb (anti-LACK IHC) was standardized with five paraffin-embedded skin biopsy samples from patients with confirmed CL, previously diagnosed by microscopy and quantitative PCR (qPCR) targeting kinetoplast DNA (kDNA). The procedure was adapted from Freire et al. (2022) [21], using the Bond Polymer Refine Red Detection (Leica Microsystems, Newcastle, UK).

Briefly, 4-µm tissue sections were mounted on ImmunoSlides (EasyPath Diagnósticos, Indaiatuba, São Paulo, Brazil) and incubated overnight at 56 °C. Dewaxing, rehydration, and antigen retrieval were performed using Trilogy® (Cell Marque, Rocklin, CA, USA) diluted 1:100 and heated in a steamer for 30 min at approximately 90 °C. The IHC protocol consisted of the following steps: (I) Blocking with 5% skim milk in PBS for 30 min; (II) Incubation with anti-LACK mAb for 60 min; (III) Application of post-primary alkaline phosphatase (AP)–conjugated antibody for 30 min; (IV) Incubation with polymer-based AP reagent for 30 min; (V) Chromogen development using the Red reagent for 3 min; and (VI) Counterstaining with hematoxylin for 3 min.

### Validation of the immunohistochemistry technique in human samples

#### Participants and samples.

A total of 104 patients with clinical suspicion of CL who attended the Municipal Polyclinic in Teófilo Otoni, Brazil, between 2019 and 2020 were included in this study. The lesioned area was cleaned with chlorhexidine and anesthetized with 2% lidocaine. Two 4-mm punch biopsies were obtained from the active border of each ulcerative lesion.

One biopsy specimen was used to prepare imprints on glass slides and was then stored in microtubes at –20 °C for DNA extraction and PCR analysis. The second biopsy specimen was fixed in 10% neutral buffered formalin and processed for histopathological (HE) and immunohistochemical (anti-LACK IHC) examinations. All samples were anonymized, and laboratory personnel were blinded to the clinical status of the samples.

#### Direct examination (DE).

Direct microscopy was performed at the *Laboratório Macroregional de Teófilo Otoni*, part of the *Rede Estadual de Laboratórios de Saúde Pública do Estado de Minas Gerais*, as part of routine CL diagnosis. Each biopsy specimen was gently pressed eight times against a glass slide to obtain tissue imprints, which were air-dried, fixed in methanol, and stained with Giemsa. Slides were examined under a light microscope at 100 × magnification. Samples were considered positive when amastigote forms were visualized in the imprints.

#### Histopathological examination (HE).

Skin lesion fragments were fixed in 10% buffered formalin (pH 7.2) for at least 24 h. The tissues were then dehydrated, cleared, and embedded in paraffin using an automatic tissue processor (PT05 TS, Lupetec, São Carlos, SP, Brazil) and an inclusion center (CI 2014, Lupetec). Sections of 4 µm were cut, mounted on glass slides, and stained with hematoxylin–eosin (HE). Slides were examined under 10 × , 40 × , and 100 × magnifications using a Zeiss microscope (Hallbergmoos, Germany) for the presence of *Leishmania* amastigotes [28].

#### Polymerase chain reaction (kDNA PCR).

Total DNA was extracted from patient biopsies using the DNeasy Blood and Tissue Kits (Qiagen, Hilden, Germany), according to the manufacturer’s instructions. Genomic DNA from *L. braziliensis* promastigotes cultured in NNN/LIT biphasic medium supplemented with 20% inactivated fetal bovine serum (FBS) and 1% Penicillin–Streptomycin was used as a positive control. DNA concentration and purity were measured with a NanoDrop spectrophotometer (GE Healthcare, Chicago, IL, USA).

PCR amplification targeted a 120-bp fragment of the *Leishmania* kinetoplast minicircle DNA using primers 150 (sense) 5′-(C/G)(C/G)(G/C)CC(C/A)CTAT(T/A)TTACACCAACCCC-3′ and 152 (antisense) 5′-GGGGAGGGGCGTTCTGCGAA-3′ [29,30]. Each 20 µL reaction contained 2 µL 10 × PCR buffer, 1.5 mM MgCl_2_, 0.5 µM of each primer (Integrated DNA Technologies, Coralville, IA, USA), 200 µM dNTPs, 20 ng of template DNA, and ultrapure water.

Amplifications were carried out in a thermocycler (ProFlex PCR System, Applied Biosystems, Foster City, CA, USA) under the following conditions: initial denaturation at 94 °C for 4 min; 35 cycles of 94 °C for 30 s, 60 °C for 30 s, and 72 °C for 30 s; and a final extension at 72 °C for 10 min. PCR reactions containing no DNA template and *L. braziliensis* DNA were used as negative and positive controls, respectively. Amplicons were resolved by 1% agarose gel electrophoresis stained with ethidium bromide, and bands were visualized using the ImageQuant LAS 4000 system (GE Healthcare, Chicago, IL, USA).

#### Anti-LACK IHC technique.

The optimized anti-LACK IHC protocol was performed using the Bond Polymer Refine Red Detection system (Leica Microsystems, Newcastle, UK) for visualization of the antigen–antibody complex. Dewaxing, rehydration, and antigen retrieval were performed in a 1:100 dilution of Trilogy® solution (Cell Marque, Rocklin, CA, USA) for 30 min at 90 °C in a steamer. Non-specific binding was blocked with PBS containing 5% skim milk for 40 min at room temperature (RT).

Slides were incubated with the anti-LACK mAb (1:50) diluted in PBS containing 1% bovine serum albumin (BSA) and 0.1% sodium azide for 60 min at RT. After washing in Tris buffer (5 mM Tris base, 140 mM NaCl, pH 7.6), slides were incubated with the post-primary alkaline phosphatase (AP) antibody for 30 min at RT, followed by incubation with the polymer-based AP reagent for another 30 min.

The chromogenic reaction was developed using the Red reagent mixture (Part A 1:10, Part B 1:50, Part C 1:50 in Part D) for 3 min. Slides were rinsed in distilled water, counterstained with hematoxylin for 3 min, mounted with Entellan® (Merck KGaA, Darmstadt, Germany), and examined under 10 × , 40 × , and 100 × magnifications using a Zeiss microscope (Hallbergmoos, Germany) to detect *Leishmania* amastigotes.

#### Database and statistical analyses.

Diagnostic performance was analyzed using MedCalc software version 15.0 (MedCalc Software, Ostend, Belgium). Comparisons between diagnostic methods were performed using the chi-square test, with a significance level of 5%. Agreement between tests was assessed using Cohen’s Kappa coefficient, interpreted according to Landis and Koch (1977): < 0 (poor), 0.00–0.20 (slight), 0.21–0.40 (fair), 0.41–0.60 (moderate), 0.61–0.80 (substantial), and 0.81–1.00 (almost perfect).

### Ethics statement

Informed written consent was obtained in accordance with the national legislation and institutional requirements, and this study was approved by the Human Research Ethics Committee of the Instituto René Rachou, Fundação Oswaldo Cruz (Comitê de Ética em Pesquisas do Instituto René Rachou/Fundação Oswaldo Cruz, CAAE No. 56188716.5.0000.5091). The use of BALB/c mice (*Mus musculus*) and golden hamsters (*Mesocricetus auratus*) followed ethical guidelines and was approved by the Animal Use Ethics Committee of Fiocruz (licenses LW-15/15 and LW-4/18).

## Results

### Production of the recombinant LACK antigen to obtain the immunogen

The 210–amino acid sequence derived from *L. braziliensis* LACK was back-translated and optimized for expression in a prokaryotic system. Successful transformation of *E. coli* BL21 Star cells with the synthetic *LACK* gene (630 bp) was confirmed by restriction digestion analysis (Fig 1a).

**Fig 1:**
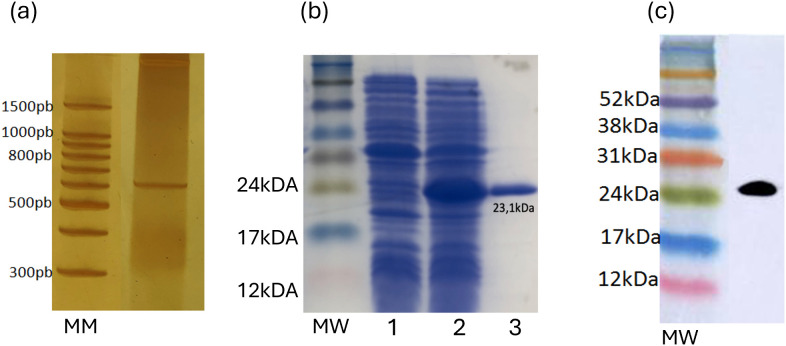
Production and characterization of recombinant LACK (rLACK). (a) Silver-stained polyacrylamide gel showing the synthetic LACK gene fragment (630 bp) after BamHI/HindIII restriction digestion; (b) 15% SDS-PAGE showing bacterial protein extracts before IPTG induction (lane 1), after IPTG induction (lane 2), and purified recombinant LACK protein (lane 3); (c) Western blot confirming expression of recombinant LACK using an anti-6 × His monoclonal antibody. MM: 100 bp DNA Ladder (Promega, USA); MW: Amersham ECL Rainbow Markers (GE Life Science, Chicago, IL, USA).

Protein expression and purification were verified by 15% SDS–PAGE, which demonstrated a distinct band corresponding to the expected molecular weight of the recombinant LACK (rLACK) protein after IPTG induction (Fig 1b). The identity of the expressed protein was further confirmed by Western blotting using an anti–6 × His mAb, which detected a single band of approximately 23 kDa (Fig 1c).

### Production of the anti-LACK monoclonal antibody

A marked anti-LACK antibody response was observed in immunized mice prior to the third immunization, with antibody titers remaining high after the fifth dose, as determined by ELISA (Fig 2a). The specificity of the anti-LACK mAb for the recombinant antigen (rLACK) was confirmed by Western blotting (Fig 2b).

**Fig 2:**
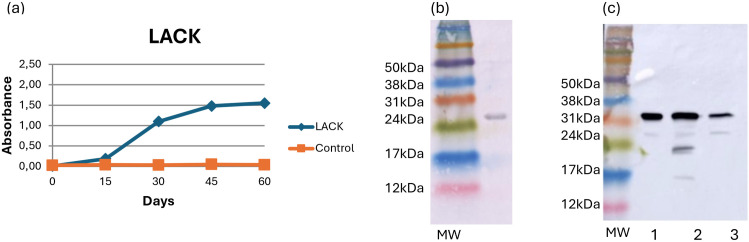
Production and characterization of the anti-LACK monoclonal antibody (mAb) and its reactivity against recombinant and native LACK antigens. (a) Kinetics of anti-LACK IgG production in mice immunized with recombinant LACK (rLACK) or saline emulsified in Freund’s adjuvant. Serum samples were collected before each immunization and analyzed by indirect ELISA. (b) Western blot demonstrating specific recognition of purified rLACK by the anti-LACK monoclonal antibody. (c) Western blot demonstrating recognition of native LACK in soluble antigen extracts from *L*. (*L*.) *amazonensis* (lane 1), *L*. (*V*.) *braziliensis* (lane 2), and *L*. (*V*.) *guyanensis* (lane 3). MW: Amersham ECL Rainbow molecular weight markers (GE Life Sciences, Chicago, IL, USA).

### Recognition of native LACK antigen to demonstrate cross-species recognition

The antibody successfully recognized the native LACK antigen in soluble *Leishmania* extracts from *L. (L.) amazonensis* (SLaA), *L. (V.) braziliensis* (SLbA), and *L. (V.) guyanensis* (SLgA), represented by a protein band of approximately 32 kDa of the full antigen sequence (Fig 2c).

### Detection of amastigote forms of *Leishmania* spp. in histological sections from experimentally infected animals to confirm tissue detection

IHC using the anti-LACK mAb successfully detected and labeled amastigote forms of *L. (L.) amazonensis*, *L. (V.) braziliensis*, and *L. (V.) guyanensis* in skin lesion samples from experimentally infected hamsters. Detection was performed using the Bond Polymer Refine Red system, resulting in clear visualization of the parasites within tissue sections (Fig 3).

**Fig 3:**
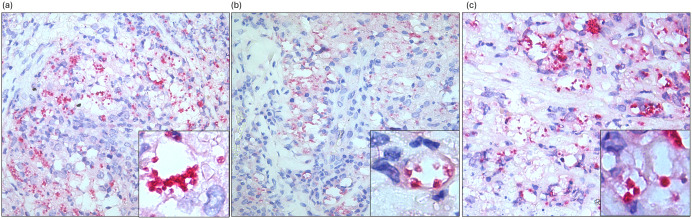
Immunohistochemical detection of *Leishmania* amastigotes in skin lesions from experimentally infected hamsters. Histological sections from hamsters infected with *L. (L.) amazonensis* (a), *L. (V.) braziliensis* (b), and *L. (V.) guyanensis* (c) stained by immunohistochemistry using the anti-LACK monoclonal antibody (mAb). Amastigote forms are specifically labeled in red by the Bond Polymer Refine Red Detection system (arrows), with representative higher-magnification insets highlighting labeled intracellular parasites.

### Standardization of the IHC protocol using anti-LACK monoclonal antibody

Optimization of blocking conditions, antibody dilution and chromogen incubation resulted in minimal background staining while maintaining strong parasite labelling. The optimized protocol (S2 File) was subsequently used for validation in human biopsy samples.

### Validation of anti-LACK IHC for diagnostic of cutaneous leishmaniasis to assess diagnostic performance

A total of 104 participants who attended the Municipal Polyclinic in Teófilo Otoni, Brazil, between 2019 and 2020 were included in this study. The kDNA-PCR assay was used as the reference standard for classifying individuals as CL cases or CL non-cases. Among the participants, 54 were confirmed CL cases by PCR, while 51 were PCR-negative individuals with suspected alternative etiologies (S1 Fig).

Of the confirmed CL cases, 11/54 participants resided in the urban area of Teófilo Otoni, and 43/54 lived in the surrounding regions, including rural communities and districts. This group comprised 23 females (median age: 42 years, range 23–49) and 31 males (median age: 53 years, range 41–61). Thirty-nine participants presented with a single lesion (0.24–15.3 mm^2^), six had two lesions (0.09–3.3 mm^2^), four had three lesions (0.19–10.5 mm^2^), and three had four lesions (0.35–10.4 mm^2^). Two patients exhibited multiple lesions for which the total area was not measured. The duration of illness ranged from 0.67 to 12 months. All CL cases were treated with meglumine antimoniate (Glucantime®, Sanofi-Aventis Farmacêutica Ltda., Suzano, SP, Brazil) in accordance with the Brazilian Ministry of Health guidelines [3].

The diagnostic performance of the anti-LACK IHC was compared with HE and DE (Table 1). The anti-LACK IHC assay showed the highest sensitivity (59.3%), followed by DE (53.7%; *p* = 0.55) and HE (42.6%; *p* = 0.08). Specificity was 98% for anti-LACK IHC and DE and 96% for HE, without statistically significant difference (*p* = 0.56). The accuracy was highest (77.9%) with anti-LAC IHC than DE (75.0%; *p* = 0.62) and HE (68.3%; *p* = 0.12). Representative photomicrographs of anti-LACK IHC and HE performed on a human skin biopsy from a confirmed CL case are presented in Fig 4.

**Table 1:** Diagnostic performance parameters of immunohistochemistry using anti-LACK monoclonal antibody (anti-LACK IHC), direct examination (DE) and histopathological examination (HE).

Diagnostic tests	Sensitivity (%) [CI95%] n	Specificity (%) n	Accuracy (%) [CI95%] n
anti-LACK IHC	59.3 [40.5–83.7] (32/54)	98 [89.5–99.7] (49/50)	77.9 [61.9–96.8] (81/104)
DE	53.7 [36–77.1] (29/54)	98 [89.5–99.6] (49/50)	75.0 [59.3–93.6] (78/104)
HE	42.6 [27–63.9] (23/54)	96 [86.5–99] (48/50)	68.3 [53.3–86.1] (71/104)

Difference in the sensitivity rates: anti-LACK IHC x DE: *p* = 0.55, anti-LACK IHC x HE: *p* = 0.08; Difference in the specificity rates: anti-LACK IHC x HE: *p* = 0.56; Difference in the accuracy rates: anti-LACK IHC x DE: *p* = 0.62, anti-LACK IHC x HE: *p* = 0.12

**Fig 4:**
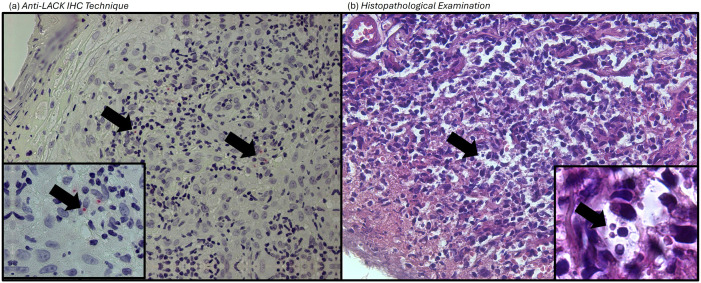
Representative detection of *Leishmania* amastigotes (40x) in a human skin biopsy from a patient with cutaneous leishmaniasis. (a) Anti-LACK immunohistochemistry (IHC) showing immunolabeled amastigote forms (red staining, black arrows). (b) Conventional histophatological examination (HE) staining of the corresponding lesion demonstrating amastigote forms within macrophages (black arrows). Insets show higher magnification of representative amastigotes (100x).

The agreement between PCR–kDNA, anti-LACK IHC, and other diagnostic techniques in classifying CL cases and non-cases is presented in Table 2. The Kappa coefficient values ranged from 0.38 to 0.56, indicating fair to moderate agreement. The lowest concordance was observed between PCR–kDNA and HE, whereas the highest agreement was found between PCR–kDNA and the anti-LACK IHC assay.

**Table 2:** Agreement analysis of results of the diagnostic techniques applied in the CL cases and non-cases.

	anti-LACK IHC			DE			HE		
	+	–	Total	+	–	Total	+	–	Total
CL cases	32	22	54	29	25	54	23	31	54
CL non-cases	1	49	50	1	49	50	2	48	50
Total	33	71	104	30	74	104	25	79	104
Kappa index	0,56 (0.42–0.71)			0,51 (0.36–0.65)			0.38 (0.23–0.52)		

DE: Direct Examination; HE: Histopathological Examination.

CL Cases and non-cases were defined by PCR assay results.

### Serial laboratory testing

The diagnosis of CL can be challenging, and in some cases, additional laboratory tests on skin biopsy specimens are required to confirm the diagnosis. To improve diagnostic performance, a sequential (combined) analysis of tests was performed (Table 3).

**Table 3:** Diagnostic performance parameters of serial laboratory testing of DE plus anti-LACK IHC and HE plus anti-LACK IHC.

Diagnostic tests	Sensitivity (%) [CI95%] n	Specificity (%) [CI95%] n	Accuracy (%) n
DE plus anti-LACK IHC	72.2 [51–99] (39/54)	96 [70.8–100] (48/50)	83.7 [67–100] (87/104)
HE plus anti-LACK IHC	64.8 [45.2–90.1] (35/54)	96 [70.8–100] (48/50)	79.8 [63.6–98.9] (83/104)

DE: Direct Examination HE: Histopathological Examination.

The combination of direct DE with the anti-LACK IHC assay increased sensitivity from 53.7% to 72.2% (*p* = 0.23), slightly reduced specificity from 98% to 96% (*p* = 0.92), and improved overall accuracy from 75.0% to 83.7% (*p* = 0.48). Similarly, combining HE with anti-LACK IHC increased sensitivity from 42.6% to 64.8% (*p* = 0.12), maintained specificity at 96%, and improved accuracy from 68.3% to 79.8% (*p* = 0.33).

The results of the combined diagnostic methods (DE plus anti-LACK IHC and HE plus anti-LACK IHC) were cross-tabulated and are presented in Table 4. When the results of DE plus anti-LACK IHC were compared with those of DE alone in relation to the CL case classification, the Kappa coefficient was 0.68, indicating substantial agreement. Similarly, the cross-tabulation of HE plus anti-LACK IHC results yielded a Kappa coefficient of 0.60, corresponding to moderate agreement.

**Table 4:** Agreement analysis of results of the diagnostic techniques used in this study.

	DE plus anti-LACK IHC			HE plus anti-LACK IHC		
	+	–	Total	+	–	Total
CL cases	39	15	54	35	19	54
CL non-cases	2	48	50	2	48	50
Total	40	5	104	37	67	104
Kappa index	0,68 (0.54–0.81)			0.60 (0.46–0.75)		

DE: Direct Examination HE: Histopathological Examination.

CL Cases and non-cases were defined by PCR assay result.

## Discussion

The diagnosis of CL remains challenging for clinicians, as its nonspecific clinical presentation overlaps with a broad spectrum of dermatological conditions, including allergic reactions to insect bites, stasis ulcers, sporotrichosis, leprosy, skin cancer, and lupus erythematosus [31]. In cases of low parasite burden, commonly observed in immunocompetent patients and in chronic lesions, parasitological methods such as direct examination and parasite culture frequently fail to detect the organism. In recent years, PCR assays targeting different *Leishmania* DNA sequences have shown high sensitivity (75.7%–100%) and specificity (up to 100%) [32]. Although biopsies are frequently performed, the diagnosis often remains inconclusive due to the limited sensitivity of conventional techniques. In this context, IHC employing monoclonal antibodies against *Leishmania* antigens represents a promising complementary strategy to enhance diagnostic accuracy, particularly when used in association with HE or DE or in settings where PCR is unavailable or not routinely performed. For several decades, the LACK protein has been investigated as a candidate for vaccine development, diagnostic testing, and as a therapeutic target [33–35]. Although the LACK sequences in *L. amazonensis*, *L. braziliensis*, and *L. guyanensis* are not completely identical, the anti-LACK mAb successfully recognized the native LACK antigen in soluble extracts and detected amastigotes in histological sections from hamsters experimentally infected with the main *Leishmania* species responsible for CL in Brazil. This finding is particularly important, as it indicates the potential applicability of this mAb across different endemic regions of the country. However, new studies still are necessary to prove its applicability in the CL diagnosis routine in different Brazilian endemic regions.

Building on these findings, the IHC technique was validated using human skin lesion samples from patients with suspected CL. The anti-LACK IHC correctly identified 32 of 54 confirmed CL cases, corresponding to a sensitivity of 59.3%. Although this value exceeded the observed sensitivities of direct examination (53.7%) and histopathology (42.6%), these differences were not statistically significant (*p* = 0.70 and *p* = 0.22, respectively). Therefore, our findings do not demonstrate superiority of anti-LACK IHC over DE or HE, but rather indicate that it achieved comparable diagnostic performance while offering the practical advantages of a standardized monoclonal antibody-based assay.

The sensitivity observed for anti-LACK IHC (59.3%) was comparable to that reported for immunohistochemical assays using polyclonal sera and biotin-based detection systems, which ranged from 58.5% to 80% [13–17,35]. Although the present study did not demonstrate higher diagnostic sensitivity than previously published IHC assays, its main contribution lies in the development and validation of a standardized monoclonal antibody-based assay. Unlike polyclonal antibodies, monoclonal antibodies recognize a single defined epitope, providing greater batch-to-batch consistency, unlimited production from a stable hybridoma clone, and improved assay standardization. These characteristics enhance the reproducibility and long-term sustainability of immunohistochemical protocols, making monoclonal antibodies more suitable for routine diagnostic practice and large-scale production. Our findings are consistent with previous studies reporting sensitivities of 51% using an anti-*L. gerbilli* monoclonal antibody [36], and slightly different from IHQ using monoclonal antibodies against *L. major* and *L. tropica* (around 90%) [18] and using a CD1a-specific monoclonal antibody (94%) [37]. However, direct comparison of sensitivity across studies should be interpreted with caution because of substantial differences in study design, reference standards, patient populations, lesion characteristics, *Leishmania* species, and IHC detection systems.

The specificity of anti-LACK IHC (98%) was comparable to that of DE (98%) and HE (96%) (*p* = 0.92). Both anti-LACK IHC and DE yielded a single false-positive result, whereas HE produced two. The high specificity observed may be partially attributed to the use of Trilogy® and Bond Polymer Refine Red reagents, which minimized background staining and nonspecific labeling. Similarly, other studies have reported high specificity rates for IHC in CL diagnosis, ranging from 94.6% to 100% when using mAbs [18,21]. However, a limitation of this study is that potential cross-reactivity with other pathogens, including *Sporothrix braziliensis*, *Mycobacterium tuberculosis*, could not be evaluated and should be investigated in future studies.

The agreement analysis demonstrated fair to moderate concordance (κ = 0.38–0.56) between kDNA-PCR and the other diagnostic methods. The lower concordance observed for anti-LACK IHC, DE, and HE primarily reflects their lower sensitivity compared with PCR, which is expected given that PCR detects parasite DNA from a substantially larger tissue sample, whereas microscopy- and histology-based methods rely on direct visualization of amastigotes in a limited amount of tissue. PCR remains the most sensitive diagnostic method evaluated in the present study and should be considered the preferred laboratory approach whenever available. Therefore, the potential role of anti-LACK IHC is not to replace molecular diagnosis but to complement tissue-based diagnostic methods in pathology laboratories or settings where PCR is unavailable.

The results of the present study demonstrated that combining DE with anti-LACK IHC increased sensitivity and accuracy without significantly reducing specificity (maintained at 96%). This sequential approach improved diagnostic accuracy from 75% to 83.7% (*p* = 0.48), detecting seven additional PCR-confirmed CL cases that had been missed by DE alone. These findings are consistent with Freire et al. (2022) [21], who reported an increase in CL positivity from 77.6% to 95.9% when combining DE with IHC. However, DE is not always available in pathology laboratories.

Similarly, combining HE with anti-LACK IHC identified 11 additional PCR-confirmed cases that were negative by HE alone, improving diagnostic accuracy from 68.3% to 79.8% (*p* = 0.33) while maintaining specificity at 96%. Other authors have also observed improved positivity rates when HE and IHC were used together [15,18]. Since both methods employ histological sections, HE relying on direct staining and IHC on immunostaining, their combined use is feasible in pathology laboratories and may enhance diagnostic yield for CL.

In summary, the findings of this study do not support the use of anti-LACK mAb IHC as a stand-alone diagnostic tool for CL. However, when applied as a complementary method, particularly in combination with DE or HE, anti-LACK mAb IHC may increase the detection of CL among tissue-based diagnostic methods while maintaining high specificity. In clinical settings where skin biopsy is performed as part of the diagnostic investigation of CL but conventional histopathological or parasitological methods fail to conclusively identify amastigotes, IHQ represents a useful tool for diagnostic confirmation. However, the development and evaluation of additional mAb targeting other *Leishmania* antigens should be encouraged to further optimize IHC-based diagnostic strategies and support timely and accurate identification of CL in routine pathology practice.

## Supporting information

S1 File Amino acid sequence of the *Leishmania* activated C kinase (A4HGX7_LEIBR), highlighting the regions reverse-translated for minigene construction. (DOCX)

S2 File Optimization of Immunohistochemistry Protocol for Detection of *Leishmania* Antigen Using Anti-LACK Monoclonal Antibody (anti-LACK IHC). (DOCX)

S1 Fig Representative agarose gel electrophoresis of kDNA PCR amplification from skin biopsy samples of 12 patients with suspected cutaneous leishmaniasis. Lanes 1–12 correspond to individual patient samples; lanes 4 is PCR-negative, whereas the remaining samples are PCR-positive, showing the expected 120-bp amplicon. MM, 100-bp DNA ladder (Promega, Madison, WI, USA); NC, no-template negative control; PC, positive control (*Leishmania braziliensis* DNA).
(TIF)
