# Supplementary material for: Anti-Leishmania activated C-kinase monoclonal antibody immunohistochemical technique for cutaneous leishmaniasis diagnosis
Source: PLoS One. 2026 Aug 5;21(8):e0339746. doi: 10.1371/journal.pone.0339746 (PMC13440861; doi:10.1371/journal.pone.0339746)
Supplement: Supplementary file 1 [file pone.0339746.s001.docx]

**Supplementary file 1.** Amino acid sequence of the *Leishmania* activated C kinase (A4HGX7_LEIBR), highlighting the regions reverse-translated for gene construction.
