# Supplementary material for: Anti-Leishmania activated C-kinase monoclonal antibody immunohistochemical technique for cutaneous leishmaniasis diagnosis
Source: PLoS One. 2026 Aug 5;21(8):e0339746. doi: 10.1371/journal.pone.0339746 (PMC13440861; doi:10.1371/journal.pone.0339746)
Supplement: Supplementary file 2 [file pone.0339746.s002.docx]

**Supplementary file 2.** Optimization of Immunohistochemistry Protocol for Detection of *Leishmania* Antigen Using Anti-LACK Monoclonal Antibody (anti-LACK IHC)

| Parameter | Conditions Tested | Final Selected | Rationale |
| --- | --- | --- | --- |
| Blocking Free Sites | 30 min, 40 min | 40 min | Reduced background staining |
| mAb Dilution | 1:500, 1:200, 1:100, 1:50 | 1:50 | Best detection |
| Subtrate/ Chromogen Dilution¹ | A(1:5); B(1:25); C (1:25) dilution in D for 3’ /  A(1:10); B(1:50); C (1:50) dilution in D for 3’ | A(1:10); B(1:50); C (1:50) dilution in D for 3’ | Balanced signal/background |

Legend: ¹Bond Polymer Refine Red Detection (Leica Microsystems, Newcastle, ENG)
