# Supplementary material for: Anti-Leishmania activated C-kinase monoclonal antibody immunohistochemical technique for cutaneous leishmaniasis diagnosis
Source: PLoS One. 2026 Aug 5;21(8):e0339746. doi: 10.1371/journal.pone.0339746 (PMC13440861; doi:10.1371/journal.pone.0339746)
Supplement: Supplementary file 3 [file pone.0339746.s004.docx]

The paper titled as” **Anti- Leishmania-activated C-kinase monoclonal antibody immunohistochemical**

**technique for cutaneous leishmaniasis diagnosis**”. This study aimed to develop and validate an immunohistochemistry (IHC) technique that employs an anti- Leishmania-activated C kinase (LACK) antigen (anti-LACK) monoclonal antibody (mAb) for the diagnosis of cutaneous leishmaniasis (CL).

**Studies on** the diagnosis of **CL** remain **important** due to ongoing challenges in accuracy, accessibility, and disease control. Current methods such as parasitological tests, often lack sensitivity because of factors like low parasite load, poor sample quality and limited technician expertise.

Major Comments:

1) This diagnostic method (IHE) requires at least a **4 mm biopsy** of the patient’s lesion, which is not practical in healthcare centers—especially for children or patients with lesions in sensitive areas of the body, such as the face. It also requires special equipment, clean rooms, and trained professionals
2) IHC techniques struggle mainly with poor reproducibility, resource shortages and technical errors from inconsistent protocols or training, especially in **low income healthcare** centers of countries where leishmaniasis is endemic.

Minor Comments:

1) Total number of the patients better to be mentioned in the **methods** section.
2) All the **figure**s and **their numbering** are not clear.
3) **Figure 1**, in “b” part: MW lane bands do not have any size.
4) It would be better to show some of positive and negative **PCR results** from the patients on an agarose gel.
