# Supplementary material for: Anti-Leishmania activated C-kinase monoclonal antibody immunohistochemical technique for cutaneous leishmaniasis diagnosis
Source: PLoS One. 2026 Aug 5;21(8):e0339746. doi: 10.1371/journal.pone.0339746 (PMC13440861; doi:10.1371/journal.pone.0339746)
Supplement: Supplementary file 4 [file pone.0339746.s006.docx]

**Comments:**

This study presents the development of an anti-LACK monoclonal antibody-based immunohistochemical (IHC) assay for the diagnosis of cutaneous leishmaniasis. While the topic is relevant, the manuscript is not currently in a suitable condition for publication. Significant revisions are required to address major concerns related to methodology, data interpretation, statistical analysis, and clarity of presentation.

**Major revision**

1. The abstract is generally well-structured and provides a comprehensive overview of the study; however, several issues affect its clarity, scientific rigour, and interpretation of results. First, there is a tendency to overstate findings despite the lack of statistical significance. Although anti-LACK IHC shows numerically higher sensitivity, specificity, and accuracy compared to DE and HE, the authors themselves report that these differences are not statistically significant. Therefore, terms such as “outperforming DE and HE” are not supported by the presented statistical evidence and should be revised to a more neutral interpretation. The abstract requires revision to ensure that conclusions are strictly aligned with statistical evidence and do not over interpret non-significant differences.
2. The Results and Materials & Methods sections are highly confusing and appear poorly coordinated. It seems that multiple redundant experiments were performed to demonstrate a single conclusion. Kindly improve the clarity and ensure that both sections correspond properly with each other.
3. In Figure 2C, the protein sizes appear highly variable. The authors should properly describe this in the Results section and ensure the figure is clearly annotated, including the relevant regions.
4. Please provide a proper flow diagram showing the hamster grouping and the challenges with different Leishmania species, as the current presentation is very confusing
5. As mentioned by the authors, the monoclonal antibody purity was assessed by 15% SDS-PAGE (lines 183–184). Is SDS-PAGE alone sufficient to determine purity (author should provide figure in the supplement), or should it be confirmed by Western blotting?
6. In the revised manuscript, the authors stated that both positive and negative PCR results are shown in the figure. However, the PCR figure lacks clear markings, and the results are unclear. Please provide a refined figure with appropriate labeling. Not only the PCR figure, but all figures require proper marking and labeling. In particular, Figure 3 is very unclear. Please provide refined figures with clear and appropriate labels. Despite the reviewer’s comments, the same errors have been repeated in the revised manuscript.
7. Where is the figure showing DE, HE and anti-LACK IHC results (10×, 40×, and 100× magnifications as mentioned in the material and method section) on human skin samples? The authors have provided the data only in tabular form, which is not sufficient for such a study. Tables are useful mainly for comparison and statistical analysis, but representative figures for DE, HE and IHC are also required, with proper identification of amastigotes. It is difficult to interpret the presence of amastigotes based only on tabular data. DE, HE and IHC results should be confirmed by clear visualization of amastigotes in human skin samples from CL cases.
8. As reported by the authors, the polyclonal antibody shows 58–80% sensitivity, and the LACK monoclonal antibody shows 59% sensitivity. In this context, what is the necessity and advantage of this study and the use of such a monoclonal antibody? The justification provided in lines 481–483 does not appear to be valid.
9. How can the authors state that “This finding is particularly important, as it indicates the potential applicability of this mAb across different endemic regions of the country”?
10. p-values are much greater than 0.05, which means the differences observed are not statistically significant. So, the author cannot claim a real difference between the methods or groups.
11. Kappa is usually used for **agreement between two independent raters/tests,** not “improvement over another method. The authors should avoid implying **diagnostic superiority** based only on Kappa,
12. The interpretation of the agreement analysis is not adequately justified. Although the authors report fair to moderate agreement between PCR–kDNA and other diagnostic methods (κ = 0.38–0.56), this statistic only reflects concordance and should not be used to infer diagnostic superiority or clinical importance. In addition, the statement that only one or two PCR-negative cases were reclassified as positive by IHC, DE, and HE actually suggests a minimal incremental diagnostic yield of these methods over PCR for case confirmation. However, this important observation is not appropriately discussed or critically interpreted. Overall, the manuscript appears to overstate the contribution of IHC, DE, and HE without a balanced evaluation of their true added diagnostic value relative to PCR.
13. In addition, the claim of high specificity is not sufficiently critically evaluated, particularly given the absence of cross-reactivity testing with clinically relevant organisms such as Sporothrix braziliensis and Mycobacterium tuberculosis, which is acknowledged but not integrated into the interpretation of diagnostic accuracy.
14. The Discussion reads more as a descriptive compilation of studies rather than a critical analysis of the present findings. A clearer structure is needed to separate: (i) what the current study truly demonstrates, (ii) what remains statistically unsupported, and (iii) how these findings realistically impact the diagnostic utility of anti-LACK IHC compared to PCR, DE, and HE.
15. Additionally, the conclusion that the assay “successfully detected the main Leishmania species responsible for CL in Brazil” is not fully supported by the presented data, as species-specific diagnostic validation is not clearly demonstrated in the clinical sample set
16. What is the significance of conducting both DE and HE experiments separately? In my opinion, performing only one experiment should be sufficient.
