# Supplementary material for: Anti-Leishmania activated C-kinase monoclonal antibody immunohistochemical technique for cutaneous leishmaniasis diagnosis
Source: PLoS One. 2026 Aug 5;21(8):e0339746. doi: 10.1371/journal.pone.0339746 (PMC13440861; doi:10.1371/journal.pone.0339746)
Supplement: Supplementary file 5 [file pone.0339746.s007.docx]

**Responses to Reviewers**

**Reviewer #1: 
1. In the introduction, since it refers to Brazilian cases, we would advice to include the previous work in the area with similar results by Sotto et al. doi.org/10.1016/0001-706X(89)90006-5.**

We thank the reviewer for the suggestion. The study by Sotto et al. (1989) has now been included in the Introduction (lines 79–81).

**2. The initial item of the Material and Methods, 'Antigen target" is more appropriate to move to introduction since it justifies the selection of LACK to obtain the antibody for the assay**.

We agree with the reviewer. The description and justification of the antigen target were moved to the Introduction (lines 87-103), where the biological relevance and diagnostic potential of LACK are now presented in a concise manner to support the rationale for its selection. The Materials and Methods section was revised accordingly.

**3. In the Material and Methods, in the description of IHC, it is not explained if the post-primary alkaline phosphatase (AP) antibody used was the same in the experimental and patients lesion samples and if the dilutions were similar in both situations.**

Immunohistochemistry performed on experimental lesion samples was conducted prior to the standardization in human samples and aimed solely to confirm the specific labeling of Leishmania amastigotes by the anti-LACK monoclonal antibody. In experimental hamster samples, the protocol followed Freire et al., with the only modification being the anti-LACK mAb dilution (1:100). During standardization in human lesion samples, blocking time and anti-LACK mAb dilution were optimized to improve staining performance, whereas no adjustment of the substrate/chromogen dilution was required. Importantly, the same post-primary alkaline phosphatase antibody and detection system were used in all experiments, namely the Bond Polymer Refine Red Detection kit (Leica Microsystems, Newcastle, UK). These clarifications have now been incorporated into the Materials and Methods (Lines 208-210)

**4. In the results, it is clear the good performance of the anti-Lack mAb to detect the three species of Leishmania (braziliensis, amazonensis and guyanensis) through assays directed to the detection of the protein, amastigote forms in experimental and patient lesions. However, it is missing some control assays to show cross reactivities against some other etiologic agents or diseases for which the diagnostic assays for leishmaniasis show crosreactivity. It would be advisable then to test against Chagas disese or Trypanosoma and Mycobacteria. Particularty, Micobacteria should be assayed since for the immunization to obtain anti-Lack Ab, it was used the Freund's complete adjuvant that usually contains heat inactivated Mycobacterium tuberculosis.**

We appreciate the reviewer’s important suggestion regarding the evaluation of potential cross-reactivity with other pathogens. However, the patients classified as negative for CL in our study were referred to specialized clinical services for investigation of other suspected conditions, and therefore confirmatory diagnoses for alternative etiologies were not available. Thus, we were able to test cross reactivity only with skin samples from participants of two another studies (DOI: 10.1590/0037-8682-0011-2018 and DOI: 10.1590/0074-02760190324), who were diagnosed with Chagas and Hansen diseases and ethical approval to do IHC technique. The IHC anti-LACK did not show marking in the parasites (Figure 1a) and bacteria (Figure 1b).

Figure 1a. Test of cross-reactivity against *Trypanosoma cruzi* in skin histological section of a patient with Chagas disease reactivation (Chagoma reactivation).

Figure 1b. Test of cross-reactivity against *Mycobacterium leprae* in skin histological section of a patient with Virchowian hanseniasis

It is important to note that Chagas disease is generally not considered a major differential diagnosis in the clinical evaluation of CL lesions. Although rare cases of cutaneous manifestations associated with *Trypanosoma* cruzi infection have been reported, these presentations are unusual and not commonly considered a diagnostic confounder in routine clinical practice. Examples of such rare cases are described in previous reports (DOI: 10.1111/j.1365-2133.1997.tb03800.x; DOI: 10.1001/archderm.139.1.104). Nevertheless, we agree that evaluating potential cross-reactivity with other pathogens, including *Sporothrix braziliensis* and *Mycobacterium tuberculosis*, would be valuable and should be addressed in future studies designed specifically for this purpose. We have now acknowledged this limitation in the revised manuscript (Lines 493 – 500)

**Reviewer#2: 
1) This diagnostic method (IHE) requires at least a 4 mm biopsy of the patient’s lesion, which is not practical in healthcare centers—especially for children or patients with lesions in sensitive areas of the body, such as the face. It also requires special equipment, clean rooms, and trained professionals**

We agree with the reviewer that IHQ requires tissue biopsy and specific infrastructure and is therefore not suitable for all cases. However, the method proposed in this study is not intended to replace first-line diagnostic approaches nor to be implemented in decentralized healthcare units. In the context of CL, skin biopsy is frequently performed as part of routine diagnostic investigation, particularly in Brazil and other endemic countries. Nevertheless, confirmation of amastigotes based solely on conventional histopathological or parasitological examination is not always possible, often resulting in inconclusive diagnoses. In this scenario, immunohistochemistry represents a useful tool for diagnostic confirmation. Thus, IHQ should be considered a complementary diagnostic approach to be applied in pathology laboratories, particularly in cases involving lesions with inconclusive results from routine diagnostic tests. These considerations have now been incorporated and better described in the Introduction and the Discussion section.

**2) IHC techniques struggle mainly with poor reproducibility, resource shortages and technical errors from inconsistent protocols or training, especially in low-income healthcare centers of countries where leishmaniasis is endemic.**

We agree with the reviewer that IHC is a technique that requires adequate infrastructure and trained personnel. However, IHC is widely and routinely employed in pathology laboratories for the diagnosis of neoplastic, infectious, and parasitic diseases, including in low- and middle-income settings. This widespread use clearly demonstrates that, when specific and well-characterized antibodies are available, and standardized protocols are followed, IHC can be applied reliably as part of the diagnostic workflow. As clarified throughout the manuscript and emphasized in the Conclusion, the present study does not propose replacing conventional parasitological diagnostic methods. Instead, our findings support IHC using mAb as a complementary diagnostic tool to conventional histopathological techniques such as HE and DE.

**Minor Comments:**
**1) Total number of the patients better to be mentioned in the methods section.**
Thank you for this suggestion. The total number of patients is already reported in the Methods section (line 233-234).

**2) All the figures and their numbering are not clear.**

The quality of all figures has been improved, and the figure numbering has been clarified accordingly.

**3) Figure 1, in “b” part: MW lane bands do not have any size.**

The molecular weight sizes have now been included in Figure 1b.

**4) It would be better to show some of positive and negative PCR results from the patients on an agarose gel.**

We have now included a representative agarose gel image showing examples of positive and negative PCR results from patient samples. This figure has been added as Supporting information file in the revised manuscript.
