# Supplementary material for: Anti-Leishmania activated C-kinase monoclonal antibody immunohistochemical technique for cutaneous leishmaniasis diagnosis
Source: PLoS One. 2026 Aug 5;21(8):e0339746. doi: 10.1371/journal.pone.0339746 (PMC13440861; doi:10.1371/journal.pone.0339746)
Supplement: Supplementary file 6 [file pone.0339746.s008.docx]

**Comments:**

This study presents the development of an anti-LACK monoclonal antibody-based immunohistochemical (IHC) assay for the diagnosis of cutaneous leishmaniasis. While the topic is relevant, the manuscript is not currently in a suitable condition for publication. Significant revisions are required to address major concerns related to methodology, data interpretation, statistical analysis, and clarity of presentation.

**Major revision**

1. The abstract is generally well-structured and provides a comprehensive overview of the study; however, several issues affect its clarity, scientific rigour, and interpretation of results. First, there is a tendency to overstate findings despite the lack of statistical significance. Although anti-LACK IHC shows numerically higher sensitivity, specificity, and accuracy compared to DE and HE, the authors themselves report that these differences are not statistically significant. Therefore, terms such as “outperforming DE and HE” are not supported by the presented statistical evidence and should be revised to a more neutral interpretation. The abstract requires revision to ensure that conclusions are strictly aligned with statistical evidence and do not over interpret non-significant differences.

Thank you for this suggestion. We have introduced a description of the respective results and statistic differences between them. Furthermore, we rewrote the abstract to align the conclusion with the results obtained. Lines 51-64.

2. The Results and Materials & Methods sections are highly confusing and appear poorly coordinated. It seems that multiple redundant experiments were performed to demonstrate a single conclusion. Kindly improve the clarity and ensure that both sections correspond properly with each other.

To improve clarity, we reorganized the manuscript so that the Results section now follows the same sequence and subheadings as the Materials and Methods section. This better aligns each experimental procedure with its corresponding findings and clarifies the rationale for each stage of the study, from recombinant antigen production and monoclonal antibody characterization to protocol optimization and clinical validation.

3. In Figure 2C, the protein sizes appear highly variable. The authors should properly describe this in the Results section and ensure the figure is clearly annotated, including the relevant regions.

Thank you for this comment. We explained that the protein band of approximately 32 kDa was taken being the complete antigen sequence. Lines 365-366.

4. Please provide a proper flow diagram showing the hamster grouping and the challenges with different Leishmania species, as the current presentation is very confusing

The purpose of this experiment was not to compare experimental groups or evaluate infection outcomes, but simply to obtain skin tissue containing amastigote forms of three *Leishmania* species for subsequent immunohistochemical evaluation of the anti-LACK monoclonal antibody. Accordingly, we have revised the Materials and Methods section to clarify that hamsters experimentally infected with L. (L.) amazonensis, L. (V.) braziliensis, or L. (V.) guyanensis were used exclusively as a source of infected tissue for the assessment of parasite detection by IHC. Because there were only three independent groups (one per Leishmania species) and no experimental intervention, randomization, treatment, or comparative animal study design, we believe that a flow diagram would not provide additional information beyond the revised description. We therefore opted to improve the clarity of the text rather than include a schematic figure (210-212)

5. As mentioned by the authors, the monoclonal antibody purity was assessed by 15% SDS-PAGE (lines 183–184). Is SDS-PAGE alone sufficient to determine purity (author should provide figure in the supplement), or should it be confirmed by Western blotting?

Thank you for punctuating this question. SDS-PAGE is the primary technique to analysis the protein purity. Western blotting would can used, but it will not improve the visualization of the antibody molecules. Furthermore, Wb is dependent on the anti-antibody conjugated type used to reveal the antigen-antibody reaction. Thus, Wb using a conjugated anti-mouse IgG (FC specific) or anti-mouse IgG (Fab specific) can reveal only heavy and light molecules from antibody, respectively.

6. In the revised manuscript, the authors stated that both positive and negative PCR results are shown in the figure. However, the PCR figure lacks clear markings, and the results are unclear. Please provide a refined figure with appropriate labeling. Not only the PCR figure, but all figures require proper marking and labeling. In particular, Figure 3 is very unclear. Please provide refined figures with clear and appropriate labels. Despite the reviewer’s comments, the same errors have been repeated in the revised manuscript.

In the revised manuscript, we carefully reviewed all figures and their legends to improve readability and interpretation. The figure legends have been expanded to provide a clearer description of each panel, and the figures have been refined with improved labeling and annotations where appropriate. In particular, the representative PCR gel has been updated to clearly identify the molecular weight marker, the expected amplicon size, the positive and negative controls, and the PCR-positive and PCR-negative samples. Figure 3 has also been improved with clearer labeling and an expanded legend to facilitate the identification of immunolabeled *Leishmania* amastigotes. We believe these modifications have substantially improved the presentation of the figures and addressed the reviewer's concerns.

7. Where is the figure showing DE, HE and anti-LACK IHC results (10×, 40×, and 100× magnifications as mentioned in the material and method section) on human skin samples? The authors have provided the data only in tabular form, which is not sufficient for such a study. Tables are useful mainly for comparison and statistical analysis, but representative figures for DE, HE and IHC are also required, with proper identification of amastigotes. It is difficult to interpret the presence of amastigotes based only on tabular data. DE, HE and IHC results should be confirmed by clear visualization of amastigotes in human skin samples from CL cases.

We have included a new figure (Figure 4) showing representative human skin biopsy sections from a confirmed cutaneous leishmaniasis case analyzed by anti-LACK IHC and histopathological examination (HE) staining, with arrows indicating representative amastigote forms. Unfortunately, representative images of the direct examination (DE) could not be included because these slides were prepared and analyzed as part of the routine diagnostic workflow at the reference laboratory, and photomicrographs were not systematically recorded at the time of diagnosis.

8. As reported by the authors, the polyclonal antibody shows 58–80% sensitivity, and the LACK monoclonal antibody shows 59% sensitivity. In this context, what is the necessity and advantage of this study and the use of such a monoclonal antibody? The justification provided in lines 481–483 does not appear to be valid.

We agree that the sensitivity of the anti-LACK monoclonal antibody is comparable to that reported for polyclonal antibody-based IHC assays. However, the main contribution of this study is not improved sensitivity, but the development and validation of a standardized monoclonal antibody-based assay. Compared with polyclonal antibodies, monoclonal antibodies provide greater batch-to-batch reproducibility, recognize a single defined epitope, and can be produced indefinitely from a stable hybridoma clone, making them more suitable for standardized routine diagnostic use. We have revised the Discussion to better emphasize these advantages and to clarify that anti-LACK IHC is intended as a complementary diagnostic tool rather than a replacement for other methods (Lines 509–519).

9. How can the authors state that “This finding is particularly important, as it indicates the potential applicability of this mAb across different endemic regions of the country”?

In this study, we demonstrated that the anti-LACK mAb was able recognize *L. amazonensis*, *L. braziliensis* and *L. guyanensis*, the main species causative of CL in Brazil. Based in these results, we believe that anti-LACK IHC can will be used for CL diagnosis across different endemic of Brazil, where these parasites maintain transmission cycle and infect humans. However, new studies still are necessary to prove its applicability in the CL diagnosis routine in different Brazilian endemic regions. Lines 507-509.

10. p-values are much greater than 0.05, which means the differences observed are not statistically significant. So, the author cannot claim a real difference between the methods or groups.

We have revised the Discussion to avoid implying a significant difference between the methods (lines 513 – 518). The text now reports the observed sensitivity values while explicitly acknowledging that these differences were not statistically significant.

11. Kappa is usually used for **agreement between two independent raters/tests,** not “improvement over another method. The authors should avoid implying **diagnostic superiority** based only on Kappa,

Thank you for your comments. During the development of a new diagnostic test, it is necessary to compare the performance of this new test to that of an existing test. Despite diagnostic accuracy receiving more attention, the agreement index plays an important role in the interpretation of the results. For example, an agreement index can determine "Go/No-Go" decision for a new diagnostic test to advance from early development into full-scale clinical validation (doi: 10.1309/AJCPRI8XPQUEAA3K). Thus, the discordant results presented by a new test in relation to the “reference test” have been frequently used for an in-depth analysis of the false-positive and false-negative results.

12. The interpretation of the agreement analysis is not adequately justified. Although the authors report fair to moderate agreement between PCR–kDNA and other diagnostic methods (κ = 0.38–0.56), this statistic only reflects concordance and should not be used to infer diagnostic superiority or clinical importance. In addition, the statement that only one or two PCR-negative cases were reclassified as positive by IHC, DE, and HE actually suggests a minimal incremental diagnostic yield of these methods over PCR for case confirmation. However, this important observation is not appropriately discussed or critically interpreted. Overall, the manuscript appears to overstate the contribution of IHC, DE, and HE without a balanced evaluation of their true added diagnostic value relative to PCR.

We agree that Kappa coefficient reflects the level of agreement between diagnostic methods and should not be interpreted as evidence of diagnostic superiority or greater clinical value. Accordingly, we have revised the manuscript to clarify, without implying that one method is diagnostically superior to another. To address this point, we revised the Discussion (lines 546-551) to explicitly acknowledge that the main potential value of anti-LACK IHC is not to replace PCR or improve case detection beyond PCR, but to serve as a complementary diagnostic tool in settings where PCR is unavailable or when histopathological evaluation is already being performed as part of the routine investigation of skin biopsies. We also moderated our conclusions throughout the manuscript to avoid overstating the diagnostic contribution of anti-LACK IHC relative to PCR.

13. In addition, the claim of high specificity is not sufficiently critically evaluated, particularly given the absence of cross-reactivity testing with clinically relevant organisms such as Sporothrix braziliensis and Mycobacterium tuberculosis, which is acknowledged but not integrated into the interpretation of diagnostic accuracy.

Thank you for your comment. This is a concern in the development of a new diagnostic test. It is important to explain that cross-reactivity with *Sporothrix braziliensis* and *Mycobacterium tuberculosis* was not evaluated in this study, because the non-CL participants were referred to specialized clinical services for confirmatory for other diseases. However, we were able to test cross reactivity only with skin samples from participants of two another studies (DOI: 10.1590/0037-8682-0011-2018 and DOI: 10.1590/0074-02760190324), who were diagnosed with Chagas and Hansen diseases and ethical approval to do IHC technique, according to preview review` comment responses. The evaluation of the potential cross-reactivity with *Sporothrix braziliensis* would be valuable and should be addressed in future studies designed specifically for this purpose. This limitation can be found in the manuscript (Lines 543-545)

14. The Discussion reads more as a descriptive compilation of studies rather than a critical analysis of the present findings. A clearer structure is needed to separate: (i) what the current study truly demonstrates, (ii) what remains statistically unsupported, and (iii) how these findings realistically impact the diagnostic utility of anti-LACK IHC compared to PCR, DE, and HE.

We have substantially revised and reorganized the Discussion to improve its structure and critical interpretation. We reduced descriptive comparison with previous studies and placed greater emphasis on interpreting the findings of the present study, clearly distinguishing statistically supported results from observations that should be interpreted with caution. We also expanded the discussion of the clinical implications of our findings to provide a more balanced and focused evaluation of the potential role of anti-LACK IHC.

15. Additionally, the conclusion that the assay “successfully detected the main Leishmania species responsible for CL in Brazil” is not fully supported by the presented data, as species-specific diagnostic validation is not clearly demonstrated in the clinical sample set

We highlight that although the anti-LACK IHC had been able to detect the main Leishmania species responsible for CL in Brazil, new study still are necessary to prove its applicability in the CL diagnosis routine in different Brazilian endemic regions

16. What is the significance of conducting both DE and HE experiments separately? In my opinion, performing only one experiment should be sufficient.

Direct examination (DE) and histopathological examination (HE) are distinct diagnostic methods that are routinely used in different clinical and laboratory settings for the diagnosis of cutaneous leishmaniasis. DE is performed on Giemsa-stained tissue imprints obtained immediately after biopsy collection and is primarily used in clinical and public health laboratories for rapid parasitological diagnosis. In contrast, HE is performed on formalin-fixed, paraffin-embedded biopsy specimens processed in pathology laboratories and provides both parasite detection and tissue histopathological evaluation. Because anti-LACK IHC is also performed on formalin-fixed, paraffin-embedded tissue sections, its potential role differs depending on the diagnostic workflow. Comparing anti-LACK IHC separately with DE and HE allows assessment of its added value in relation to each of these established tissue-based diagnostic approaches and reflects the different clinical scenarios in which they are used. We have clarified this rationale in the introduction (lines 80-85).
