# Supplementary material for: Anti-Leishmania activated C-kinase monoclonal antibody immunohistochemical technique for cutaneous leishmaniasis diagnosis
Source: PLoS One. 2026 Aug 5;21(8):e0339746. doi: 10.1371/journal.pone.0339746 (PMC13440861; doi:10.1371/journal.pone.0339746)
Supplement: Peer review history

# Peer review: Decision Letter 0

**DOI:** 10.1371/journal.pone.0339746.r001

**Authors:** Elham Kazemirad

17 Feb 2026

PONE-D-25-66089

Anti- Leishmania-activated C-kinase monoclonal antibody immunohistochemical technique for cutaneous leishmaniasis diagnosis

PLOS One

Dear Dr. Oliveira,

Thank you for submitting your manuscript to PLOS ONE. After careful consideration, we feel that it has merit but does not fully meet PLOS ONE’s publication criteria as it currently stands. Therefore, we invite you to submit a revised version of the manuscript that addresses the points raised during the review process.

Please revise all figures and captions, recreate graphs in GraphPad Prism rather than Excel, and ensure adherence to PLOS style guidelines.

Please submit your revised manuscript by Apr 03 2026 11:59PM. If you will need more time than this to complete your revisions, please reply to this message or contact the journal office at plosone@plos.org. When you're ready to submit your revision, log on to https://www.editorialmanager.com/pone/ and select the 'Submissions Needing Revision' folder to locate your manuscript file.

Please include the following items when submitting your revised manuscript:

- A letter that responds to each point raised by the academic editor and reviewer(s). You should upload this letter as a separate file labeled 'Response to Reviewers'.
- A marked-up copy of your manuscript that highlights changes made to the original version. You should upload this as a separate file labeled 'Revised Manuscript with Track Changes'.
- An unmarked version of your revised paper without tracked changes. You should upload this as a separate file labeled 'Manuscript'.

If you would like to make changes to your financial disclosure, please include your updated statement in your cover letter. Guidelines for resubmitting your figure files are available below the reviewer comments at the end of this letter.

If applicable, we recommend that you deposit your laboratory protocols in protocols.io to enhance the reproducibility of your results. Protocols.io assigns your protocol its own identifier (DOI) so that it can be cited independently in the future. For instructions see: https://journals.plos.org/plosone/s/submission-guidelines#loc-laboratory-protocols. Additionally, PLOS ONE offers an option for publishing peer-reviewed Lab Protocol articles, which describe protocols hosted on protocols.io. Read more information on sharing protocols at https://plos.org/protocols?utm_medium=editorial-email&utm_source=authorletters&utm_campaign=protocols.

We look forward to receiving your revised manuscript.

Kind regards,

Elham Kazemirad, Ph.D

Academic Editor

PLOS One

Journal Requirements:

When submitting your revision, we need you to address these additional requirements.

1. Please ensure that your manuscript meets PLOS ONE's style requirements, including those for file naming. The PLOS ONE style templates can be found at

https://journals.plos.org/plosone/s/file?id=wjVg/PLOSOne_formatting_sample_main_body.pdf and

https://journals.plos.org/plosone/s/file?id=ba62/PLOSOne_formatting_sample_title_authors_affiliations.pdf

2. Thank you for stating the following in the Acknowledgments Section of your manuscript:

“We are grateful to Fernanda Oliveira Rodrigues for assistance with the histological secƟons. EO is grateful to CNPq-Brazil (Conselho Nacional de Desenvolvimento Cienơfico e Tecnológico) for fellowships (Proc. 301555/2022-2)”

We note that you have provided additional information within the Acknowledgements Section that is not currently declared in your Funding Statement. Please note that funding information should not appear in the Acknowledgments section or other areas of your manuscript. We will only publish funding information present in the Funding Statement section of the online submission form.

Please remove any funding-related text from the manuscript and let us know how you would like to update your Funding Statement. Currently, your Funding Statement reads as follows:

“This study was supported by the Fundação de Amparo à Pesquisa do Estado de Minas Gerais (Fapemig) – Grant APQ-02248-18, Grant APQ-04991-23, and by the Instituto René Rachou, Oswaldo Cruz Foundation”

Please include your amended statements within your cover letter; we will change the online submission form on your behalf.

3. PLOS ONE now requires that authors provide the original uncropped and unadjusted images underlying all blot or gel results reported in a submission’s figures or Supporting Information files. This policy and the journal’s other requirements for blot/gel reporting and figure preparation are described in detail at https://journals.plos.org/plosone/s/figures#loc-blot-and-gel-reporting-requirements and https://journals.plos.org/plosone/s/figures#loc-preparing-figures-from-image-files. When you submit your revised manuscript, please ensure that your figures adhere fully to these guidelines and provide the original underlying images for all blot or gel data reported in your submission. See the following link for instructions on providing the original image data: https://journals.plos.org/plosone/s/figures#loc-original-images-for-blots-and-gels.

In your cover letter, please note whether your blot/gel image data are in Supporting Information or posted at a public data repository, provide the repository URL if relevant, and provide specific details as to which raw blot/gel images, if any, are not available. Email us at plosone@plos.org if you have any questions.

4. We note that you have included the phrase “data not shown” in your manuscript. Unfortunately, this does not meet our data sharing requirements. PLOS does not permit references to inaccessible data. We require that authors provide all relevant data within the paper, Supporting Information files, or in an acceptable, public repository. Please add a citation to support this phrase or upload the data that corresponds with these findings to a stable repository (such as Figshare or Dryad) and provide and URLs, DOIs, or accession numbers that may be used to access these data. Or, if the data are not a core part of the research being presented in your study, we ask that you remove the phrase that refers to these data.

5. Please include your full ethics statement in the ‘Methods’ section of your manuscript file. In your statement, please include the full name of the IRB or ethics committee who approved or waived your study, as well as whether or not you obtained informed written or verbal consent. If consent was waived for your study, please include this information in your statement as well

6. If the reviewer comments include a recommendation to cite specific previously published works, please review and evaluate these publications to determine whether they are relevant and should be cited. There is no requirement to cite these works unless the editor has indicated otherwise.

[Note: HTML markup is below. Please do not edit.]

Reviewers' comments:

Reviewer's Responses to Questions

**Comments to the Author**

1. Is the manuscript technically sound, and do the data support the conclusions?

The manuscript must describe a technically sound piece of scientific research with data that supports the conclusions. Experiments must have been conducted rigorously, with appropriate controls, replication, and sample sizes. The conclusions must be drawn appropriately based on the data presented.

Reviewer #1: Yes

Reviewer #2: Yes

**********

2. Has the statistical analysis been performed appropriately and rigorously?

Reviewer #1: Yes

Reviewer #2: I Don't Know

**********

3. Have the authors made all data underlying the findings in their manuscript fully available?

The PLOS Data policy requires authors to make all data underlying the findings described in their manuscript fully available without restriction, with rare exception (please refer to the Data Availability Statement in the manuscript PDF file). The data should be provided as part of the manuscript or its supporting information, or deposited to a public repository. For example, in addition to summary statistics, the data points behind means, medians and variance measures should be available. If there are restrictions on publicly sharing data—e.g. participant privacy or use of data from a third party—those must be specified.

Reviewer #1: Yes

Reviewer #2: Yes

**********

4. Is the manuscript presented in an intelligible fashion and written in standard English?

PLOS ONE does not copyedit accepted manuscripts, so the language in submitted articles must be clear, correct, and unambiguous. Any typographical or grammatical errors should be corrected at revision, so please note any specific errors here.

Reviewer #1: Yes

Reviewer #2: Yes

**********

5. Review Comments to the Author

Please use the space provided to explain your answers to the questions above. You may also include additional comments for the author, including concerns about dual publication, research ethics, or publication ethics. (Please upload your review as an attachment if it exceeds 20,000 characters)

Reviewer #1: The MS presents interesting data showing good performance of the immunohistochemical detection of Leishmania amastigotes in tissue samples from exprimental and human cutaneous leishmanaisis (CL) that may contribute to improve the diagnosis of CL caused by three main species of Leishmania predominant in Brazil.

In the introduction, since it refers to Brazilian cases, we would advice to include the previous work in the area with similar results by Sotto et al. doi.org/10.1016/0001-706X(89)90006-5. The initial item of the Material and Methods, 'Antigen target" is more appropriate to move to introduction since it justifies the selection of LACK to obtain the antibody for the assay.

In the Material and Methods, in the description of IHC, it is not explained if the post-primary alkaline phosphatase (AP) antibody used was the same in the experimental and patients lesion samples and if the dilutions were similar in both situations.

In the results, it is clear the good performance of the anti-Lack mAb to detect the three species of Leishmania (braziliensis, amazonensis and guyanensis) through assays directed to the detection of the protein, amastigote forms in experimental and patient lesions. However, it is missing some control assays to show cross reactivities against some other etiologic agents or diseases for which the diagnostic assays for leishmaniasis show crosreactivity. It would be advisable then to test against Chagas disese or Trypanosoma and Mycobacteria. Particularty, Micobacteria should be assayed since for the immunization to obtain anti-Lack Ab, it was used the Freund's complete adjuvant that usually contains heat inactivated Mycobacterium tuberculosis.

Reviewer #2: The paper titled as” Anti- Leishmania-activated C-kinase monoclonal antibody immunohistochemical

technique for cutaneous leishmaniasis diagnosis”. This study aimed to develop and validate an immunohistochemistry (IHC) technique that employs an anti- Leishmania-activated C kinase (LACK) antigen (anti-LACK) monoclonal antibody (mAb) for the diagnosis of cutaneous leishmaniasis (CL).

Studies on the diagnosis of CL remain important due to ongoing challenges in accuracy, accessibility, and disease control. Current methods such as parasitological tests, often lack sensitivity because of factors like low parasite load, poor sample quality and limited technician expertise.

Major Comments:

1) This diagnostic method (IHE) requires at least a 4 mm biopsy of the patient’s lesion, which is not practical in healthcare centers—especially for children or patients with lesions in sensitive areas of the body, such as the face. It also requires special equipment, clean rooms, and trained professionals

2) IHC techniques struggle mainly with poor reproducibility, resource shortages and technical errors from inconsistent protocols or training, especially in low income healthcare centers of countries where leishmaniasis is endemic.

Minor Comments:

1) Total number of the patients better to be mentioned in the methods section.

2) All the figures and their numbering are not clear.

3) Figure 1, in “b” part: MW lane bands do not have any size.

4) It would be better to show some of positive and negative PCR results from the patients on an agarose gel.

**********

6. PLOS authors have the option to publish the peer review history of their article (what does this mean?). If published, this will include your full peer review and any attached files.

If you choose “no”, your identity will remain anonymous but your review may still be made public.

**Do you want your identity to be public for this peer review?**  For information about this choice, including consent withdrawal, please see our Privacy Policy.

Reviewer #1: **Yes:** Hiro Goto

Reviewer #2: No

**********

[NOTE: If reviewer comments were submitted as an attachment file, they will be attached to this email and accessible via the submission site. Please log into your account, locate the manuscript record, and check for the action link "View Attachments". If this link does not appear, there are no attachment files.]

To ensure your figures meet our technical requirements, please review our figure guidelines: https://journals.plos.org/plosone/s/figures

You may also use PLOS’s free figure tool, NAAS, to help you prepare publication quality figures: https://journals.plos.org/plosone/s/figures#loc-tools-for-figure-preparation.

NAAS will assess whether your figures meet our technical requirements by comparing each figure against our figure specifications.

Attachment Submitted filename: 2.1.2026 PLOS review.docx

# Author comment: Author response to Decision Letter 1

**DOI:** 10.1371/journal.pone.0339746.r002

1 Apr 2026

Responses to Reviewers

Reviewer #1:

1. In the introduction, since it refers to Brazilian cases, we would advice to include the previous work in the area with similar results by Sotto et al. doi.org/10.1016/0001-706X(89)90006-5.

We thank the reviewer for the suggestion. The study by Sotto et al. (1989) has now been included in the Introduction (lines 79–81).

2. The initial item of the Material and Methods, 'Antigen target" is more appropriate to move to introduction since it justifies the selection of LACK to obtain the antibody for the assay.

We agree with the reviewer. The description and justification of the antigen target were moved to the Introduction (lines 87-103), where the biological relevance and diagnostic potential of LACK are now presented in a concise manner to support the rationale for its selection. The Materials and Methods section was revised accordingly.

3. In the Material and Methods, in the description of IHC, it is not explained if the post-primary alkaline phosphatase (AP) antibody used was the same in the experimental and patients lesion samples and if the dilutions were similar in both situations.

Immunohistochemistry performed on experimental lesion samples was conducted prior to the standardization in human samples and aimed solely to confirm the specific labeling of Leishmania amastigotes by the anti-LACK monoclonal antibody. In experimental hamster samples, the protocol followed Freire et al., with the only modification being the anti-LACK mAb dilution (1:100). During standardization in human lesion samples, blocking time and anti-LACK mAb dilution were optimized to improve staining performance, whereas no adjustment of the substrate/chromogen dilution was required. Importantly, the same post-primary alkaline phosphatase antibody and detection system were used in all experiments, namely the Bond Polymer Refine Red Detection kit (Leica Microsystems, Newcastle, UK). These clarifications have now been incorporated into the Materials and Methods (Lines 208-210)

4. In the results, it is clear the good performance of the anti-Lack mAb to detect the three species of Leishmania (braziliensis, amazonensis and guyanensis) through assays directed to the detection of the protein, amastigote forms in experimental and patient lesions. However, it is missing some control assays to show cross reactivities against some other etiologic agents or diseases for which the diagnostic assays for leishmaniasis show crosreactivity. It would be advisable then to test against Chagas disese or Trypanosoma and Mycobacteria. Particularty, Micobacteria should be assayed since for the immunization to obtain anti-Lack Ab, it was used the Freund's complete adjuvant that usually contains heat inactivated Mycobacterium tuberculosis.

We appreciate the reviewer’s important suggestion regarding the evaluation of potential cross-reactivity with other pathogens. However, the patients classified as negative for CL in our study were referred to specialized clinical services for investigation of other suspected conditions, and therefore confirmatory diagnoses for alternative etiologies were not available. Thus, we were able to test cross reactivity only with skin samples from participants of two another studies (DOI: 10.1590/0037-8682-0011-2018 and DOI: 10.1590/0074-02760190324), who were diagnosed with Chagas and Hansen diseases and ethical approval to do IHC technique. The IHC anti-LACK did not show marking in the parasites.

It is important to note that Chagas disease is generally not considered a major differential diagnosis in the clinical evaluation of CL lesions. Although rare cases of cutaneous manifestations associated with Trypanosoma cruzi infection have been reported, these presentations are unusual and not commonly considered a diagnostic confounder in routine clinical practice. Examples of such rare cases are described in previous reports (DOI: 10.1111/j.1365-2133.1997.tb03800.x; DOI: 10.1001/archderm.139.1.104). Nevertheless, we agree that evaluating potential cross-reactivity with other pathogens, including Sporothrix braziliensis and Mycobacterium tuberculosis, would be valuable and should be addressed in future studies designed specifically for this purpose. We have now acknowledged this limitation in the revised manuscript (Lines 493 – 500)

Reviewer#2:

1) This diagnostic method (IHE) requires at least a 4 mm biopsy of the patient’s lesion, which is not practical in healthcare centers—especially for children or patients with lesions in sensitive areas of the body, such as the face. It also requires special equipment, clean rooms, and trained professionals

We agree with the reviewer that IHQ requires tissue biopsy and specific infrastructure and is therefore not suitable for all cases. However, the method proposed in this study is not intended to replace first-line diagnostic approaches nor to be implemented in decentralized healthcare units. In the context of CL, skin biopsy is frequently performed as part of routine diagnostic investigation, particularly in Brazil and other endemic countries. Nevertheless, confirmation of amastigotes based solely on conventional histopathological or parasitological examination is not always possible, often resulting in inconclusive diagnoses. In this scenario, immunohistochemistry represents a useful tool for diagnostic confirmation. Thus, IHQ should be considered a complementary diagnostic approach to be applied in pathology laboratories, particularly in cases involving lesions with inconclusive results from routine diagnostic tests. These considerations have now been incorporated and better described in the Introduction and the Discussion section.

2) IHC techniques struggle mainly with poor reproducibility, resource shortages and technical errors from inconsistent protocols or training, especially in low-income healthcare centers of countries where leishmaniasis is endemic.

We agree with the reviewer that IHC is a technique that requires adequate infrastructure and trained personnel. However, IHC is widely and routinely employed in pathology laboratories for the diagnosis of neoplastic, infectious, and parasitic diseases, including in low- and middle-income settings. This widespread use clearly demonstrates that, when specific and well-characterized antibodies are available, and standardized protocols are followed, IHC can be applied reliably as part of the diagnostic workflow. As clarified throughout the manuscript and emphasized in the Conclusion, the present study does not propose replacing conventional parasitological diagnostic methods. Instead, our findings support IHC using mAb as a complementary diagnostic tool to conventional histopathological techniques such as HE and DE.

Minor Comments:

1) Total number of the patients better to be mentioned in the methods section.

Thank you for this suggestion. The total number of patients is already reported in the Methods section (line 233-234).

2) All the figures and their numbering are not clear.

The quality of all figures has been improved, and the figure numbering has been clarified accordingly.

3) Figure 1, in “b” part: MW lane bands do not have any size.

The molecular weight sizes have now been included in Figure 1b.

4) It would be better to show some of positive and negative PCR results from the patients on an agarose gel.

We have now included a representative agarose gel image showing examples of positive and negative PCR results from patient samples. This figure has been added as Supporting information file in the revised manuscript.

Attachment Submitted filename: Responses to Reviewers.docx

# Peer review: Decision Letter 1

**DOI:** 10.1371/journal.pone.0339746.r003

**Authors:** Vikash Dubey

1 Jun 2026

PONE-D-25-66089R1

Anti- Leishmania-activated C-kinase monoclonal antibody immunohistochemical technique for cutaneous leishmaniasis diagnosis

PLOS One

Dear Dr. Oliveira,

Thank you for submitting your manuscript to PLOS ONE. After careful consideration, we feel that it has merit but does not fully meet PLOS ONE’s publication criteria as it currently stands. Therefore, we invite you to submit a revised version of the manuscript that addresses the points raised during the review process.

Please submit your revised manuscript by Jul 16 2026 11:59PM. If you will need more time than this to complete your revisions, please reply to this message or contact the journal office at plosone@plos.org. When you're ready to submit your revision, log on to https://www.editorialmanager.com/pone/ and select the 'Submissions Needing Revision' folder to locate your manuscript file.

Please include the following items when submitting your revised manuscript:

- A letter that responds to each point raised by the academic editor and reviewer(s). You should upload this letter as a separate file labeled 'Response to Reviewers'.
- A marked-up copy of your manuscript that highlights changes made to the original version. You should upload this as a separate file labeled 'Revised Manuscript with Track Changes'.
- An unmarked version of your revised paper without tracked changes. You should upload this as a separate file labeled 'Manuscript'.

If you would like to make changes to your financial disclosure, please include your updated statement in your cover letter. Guidelines for resubmitting your figure files are available below the reviewer comments at the end of this letter.

If applicable, we recommend that you deposit your laboratory protocols in protocols.io to enhance the reproducibility of your results. Protocols.io assigns your protocol its own identifier (DOI) so that it can be cited independently in the future. For instructions see: https://journals.plos.org/plosone/s/submission-guidelines#loc-laboratory-protocols. Additionally, PLOS ONE offers an option for publishing peer-reviewed Lab Protocol articles, which describe protocols hosted on protocols.io. Read more information on sharing protocols at https://plos.org/protocols?utm_medium=editorial-email&utm_source=authorletters&utm_campaign=protocols.

As the corresponding author, your ORCID iD is verified in the submission system and will appear in the published article. PLOS supports the use of ORCID, and we encourage all coauthors to register for an ORCID iD and use it as well. Please encourage your coauthors to verify their ORCID iD within the submission system before final acceptance, as unverified ORCID iDs will not appear in the published article. *Only*  the individual author can complete the verification step; PLOS staff *cannot*  verify ORCID iDs on behalf of authors.

We look forward to receiving your revised manuscript.

Kind regards,

Vikash Kumar Dubey

Academic Editor

PLOS One

Journal Requirements:

If the reviewer comments include a recommendation to cite specific previously published works, please review and evaluate these publications to determine whether they are relevant and should be cited. There is no requirement to cite these works unless the editor has indicated otherwise.

[Note: HTML markup is below. Please do not edit.]

Reviewers' comments:

Reviewer's Responses to Questions

**Comments to the Author**

1. If the authors have adequately addressed your comments raised in a previous round of review and you feel that this manuscript is now acceptable for publication, you may indicate that here to bypass the “Comments to the Author” section, enter your conflict of interest statement in the “Confidential to Editor” section, and submit your "Accept" recommendation.

Reviewer #1: All comments have been addressed

Reviewer #3: (No Response)

**********

2. Is the manuscript technically sound, and do the data support the conclusions?

The manuscript must describe a technically sound piece of scientific research with data that supports the conclusions. Experiments must have been conducted rigorously, with appropriate controls, replication, and sample sizes. The conclusions must be drawn appropriately based on the data presented.

Reviewer #1: Yes

Reviewer #3: Partly

**********

3. Has the statistical analysis been performed appropriately and rigorously?

Reviewer #1: Yes

Reviewer #3: No

**********

4. Have the authors made all data underlying the findings in their manuscript fully available?

The PLOS Data policy requires authors to make all data underlying the findings described in their manuscript fully available without restriction, with rare exception (please refer to the Data Availability Statement in the manuscript PDF file). The data should be provided as part of the manuscript or its supporting information, or deposited to a public repository. For example, in addition to summary statistics, the data points behind means, medians and variance measures should be available. If there are restrictions on publicly sharing data—e.g. participant privacy or use of data from a third party—those must be specified.

Reviewer #1: Yes

Reviewer #3: (No Response)

**********

5. Is the manuscript presented in an intelligible fashion and written in standard English?

PLOS ONE does not copyedit accepted manuscripts, so the language in submitted articles must be clear, correct, and unambiguous. Any typographical or grammatical errors should be corrected at revision, so please note any specific errors here.

Reviewer #1: Yes

Reviewer #3: Yes

**********

6. Review Comments to the Author

Please use the space provided to explain your answers to the questions above. You may also include additional comments for the author, including concerns about dual publication, research ethics, or publication ethics. (Please upload your review as an attachment if it exceeds 20,000 characters)

Reviewer #1: (No Response)

Reviewer #3: (No Response)

**********

7. PLOS authors have the option to publish the peer review history of their article (what does this mean?). If published, this will include your full peer review and any attached files.

If you choose “no”, your identity will remain anonymous but your review may still be made public.

**Do you want your identity to be public for this peer review?**  For information about this choice, including consent withdrawal, please see our Privacy Policy.

Reviewer #1: **Yes:** Hiro Goto

Reviewer #3: No

**********

[NOTE: If reviewer comments were submitted as an attachment file, they will be attached to this email and accessible via the submission site. Please log into your account, locate the manuscript record, and check for the action link "View Attachments". If this link does not appear, there are no attachment files.]

To ensure your figures meet our technical requirements, please review our figure guidelines: https://journals.plos.org/plosone/s/figures

You may also use PLOS’s free figure tool, NAAS, to help you prepare publication quality figures: https://journals.plos.org/plosone/s/figures#loc-tools-for-figure-preparation.

NAAS will assess whether your figures meet our technical requirements by comparing each figure against our figure specifications.

Attachment Submitted filename: Comments_PONE-D-25-66089_R1.docx

# Author comment: Author response to Decision Letter 2

**DOI:** 10.1371/journal.pone.0339746.r004

7 Jul 2026

Dear Editor,

We have received the reviewer’ comments regarding our manuscript PONE-D-25-66089, entitled “Anti-Leishmania-activated C-kinase monoclonal antibody immunohistochemical technique for cutaneous leishmaniasis diagnosis” submitted to the PLoS One. We have worked thoroughly to answer all queries to improve the quality of our manuscript and make it suitable to be published in PLoS One. We are presenting below all the queries stated by your considerations. The changes are highlighted in the file Manuscript IHQ_LACK trackchanges.docx to make it easier for the Reviewers/Editors to access them. We have prepared below a list that responds to the Editor and Reviewer’s comments.

Editor`requirements:

The funding statements were edited, and we would like to update for:

This study was supported by the Fundação de Amparo à Pesquisa do Estado de Minas Gerais

(Fapemig)- Grant APQ-02248-18, by the Instituto René Rachou, Fundação Oswaldo Cruz,

Fiocruz, and by the Coordenação de Aperfeiçoamento de Pessoal de Nível Superior – Brazil

(CAPES) Finance Code 001. This publication was supported by the Fundação de Amparo à

Pesquisa do Estado de Minas Gerais (Fapemig). EO is supported by fellowship of the CNPq-Brazil (Conselho Nacional de Desenvolvimento Científico e Tecnológico; Proc. 301555/2022-2).

Responses to Reviewer

1. The abstract is generally well-structured and provides a comprehensive overview of the study; however, several issues affect its clarity, scientific rigour, and interpretation of results. First, there is a tendency to overstate findings despite the lack of statistical significance. Although anti-LACK IHC shows numerically higher sensitivity, specificity, and accuracy compared to DE and HE, the authors themselves report that these differences are not statistically significant. Therefore, terms such as “outperforming DE and HE” are not supported by the presented statistical evidence and should be revised to a more neutral interpretation. The abstract requires revision to ensure that conclusions are strictly aligned with statistical evidence and do not over interpret non-significant differences.

Thank you for this suggestion. We have introduced a description of the respective results and statistic differences between them. Furthermore, we rewrote the abstract to align the conclusion with the results obtained. Lines 51-64.

2. The Results and Materials & Methods sections are highly confusing and appear poorly coordinated. It seems that multiple redundant experiments were performed to demonstrate a single conclusion. Kindly improve the clarity and ensure that both sections correspond properly with each other.

To improve clarity, we reorganized the manuscript so that the Results section now follows the same sequence and subheadings as the Materials and Methods section. This better aligns each experimental procedure with its corresponding findings and clarifies the rationale for each stage of the study, from recombinant antigen production and monoclonal antibody characterization to protocol optimization and clinical validation.

3. In Figure 2C, the protein sizes appear highly variable. The authors should properly describe this in the Results section and ensure the figure is clearly annotated, including the relevant regions.

Thank you for this comment. We explained that the protein band of approximately 32 kDa was taken being the complete antigen sequence. Lines 365-366.

4. Please provide a proper flow diagram showing the hamster grouping and the challenges with different Leishmania species, as the current presentation is very confusing

The purpose of this experiment was not to compare experimental groups or evaluate infection outcomes, but simply to obtain skin tissue containing amastigote forms of three Leishmania species for subsequent immunohistochemical evaluation of the anti-LACK monoclonal antibody. Accordingly, we have revised the Materials and Methods section to clarify that hamsters experimentally infected with L. (L.) amazonensis, L. (V.) braziliensis, or L. (V.) guyanensis were used exclusively as a source of infected tissue for the assessment of parasite detection by IHC. Because there were only three independent groups (one per Leishmania species) and no experimental intervention, randomization, treatment, or comparative animal study design, we believe that a flow diagram would not provide additional information beyond the revised description. We therefore opted to improve the clarity of the text rather than include a schematic figure (210-212)

5. As mentioned by the authors, the monoclonal antibody purity was assessed by 15% SDS-PAGE (lines 183–184). Is SDS-PAGE alone sufficient to determine purity (author should provide figure in the supplement), or should it be confirmed by Western blotting?

Thank you for punctuating this question. SDS-PAGE is the primary technique to analysis the protein purity. Western blotting would can used, but it will not improve the visualization of the antibody molecules. Furthermore, Wb is dependent on the anti-antibody conjugated type used to reveal the antigen-antibody reaction. Thus, Wb using a conjugated anti-mouse IgG (FC specific) or anti-mouse IgG (Fab specific) can reveal only heavy and light molecules from antibody, respectively.

6. In the revised manuscript, the authors stated that both positive and negative PCR results are shown in the figure. However, the PCR figure lacks clear markings, and the results are unclear. Please provide a refined figure with appropriate labeling. Not only the PCR figure, but all figures require proper marking and labeling. In particular, Figure 3 is very unclear. Please provide refined figures with clear and appropriate labels. Despite the reviewer’s comments, the same errors have been repeated in the revised manuscript.

In the revised manuscript, we carefully reviewed all figures and their legends to improve readability and interpretation. The figure legends have been expanded to provide a clearer description of each panel, and the figures have been refined with improved labeling and annotations where appropriate. In particular, the representative PCR gel has been updated to clearly identify the molecular weight marker, the expected amplicon size, the positive and negative controls, and the PCR-positive and PCR-negative samples. Figure 3 has also been improved with clearer labeling and an expanded legend to facilitate the identification of immunolabeled Leishmania amastigotes. We believe these modifications have substantially improved the presentation of the figures and addressed the reviewer's concerns.

7. Where is the figure showing DE, HE and anti-LACK IHC results (10×, 40×, and 100× magnifications as mentioned in the material and method section) on human skin samples? The authors have provided the data only in tabular form, which is not sufficient for such a study. Tables are useful mainly for comparison and statistical analysis, but representative figures for DE, HE and IHC are also required, with proper identification of amastigotes. It is difficult to interpret the presence of amastigotes based only on tabular data. DE, HE and IHC results should be confirmed by clear visualization of amastigotes in human skin samples from CL cases.

We have included a new figure (Figure 4) showing representative human skin biopsy sections from a confirmed cutaneous leishmaniasis case analyzed by anti-LACK IHC and histopathological examination (HE) staining, with arrows indicating representative amastigote forms. Unfortunately, representative images of the direct examination (DE) could not be included because these slides were prepared and analyzed as part of the routine diagnostic workflow at the reference laboratory, and photomicrographs were not systematically recorded at the time of diagnosis.

8. As reported by the authors, the polyclonal antibody shows 58–80% sensitivity, and the LACK monoclonal antibody shows 59% sensitivity. In this context, what is the necessity and advantage of this study and the use of such a monoclonal antibody? The justification provided in lines 481–483 does not appear to be valid.

We agree that the sensitivity of the anti-LACK monoclonal antibody is comparable to that reported for polyclonal antibody-based IHC assays. However, the main contribution of this study is not improved sensitivity, but the development and validation of a standardized monoclonal antibody-based assay. Compared with polyclonal antibodies, monoclonal antibodies provide greater batch-to-batch reproducibility, recognize a single defined epitope, and can be produced indefinitely from a stable hybridoma clone, making them more suitable for standardized routine diagnostic use. We have revised the Discussion to better emphasize these advantages and to clarify that anti-LACK IHC is intended as a complementary diagnostic tool rather than a replacement for other methods (Lines 509–519).

9. How can the authors state that “This finding is particularly important, as it indicates the potential applicability of this mAb across different endemic regions of the country”?

In this study, we demonstrated that the anti-LACK mAb was able recognize L. amazonensis, L. braziliensis and L. guyanensis, the main species causative of CL in Brazil. Based in these results, we believe that anti-LACK IHC can will be used for CL diagnosis across different endemic of Brazil, where these parasites maintain transmission cycle and infect humans. However, new studies still are necessary to prove its applicability in the CL diagnosis routine in different Brazilian endemic regions. Lines 507-509.

10. p-values are much greater than 0.05, which means the differences observed are not statistically significant. So, the author cannot claim a real difference between the methods or groups.

We have revised the Discussion to avoid implying a significant difference between the methods (lines 513 – 518). The text now reports the observed sensitivity values while explicitly acknowledging that these differences were not statistically significant.

11. Kappa is usually used for agreement between two independent raters/tests, not “improvement over another method. The authors should avoid implying diagnostic superiority based only on Kappa,

Thank you for your comments. During the development of a new diagnostic test, it is necessary to compare the performance of this new test to that of an existing test. Despite diagnostic accuracy receiving more attention, the agreement index plays an important role in the interpretation of the results. For example, an agreement index can determine "Go/No-Go" decision for a new diagnostic test to advance from early development into full-scale clinical validation (doi: 10.1309/AJCPRI8XPQUEAA3K). Thus, the discordant results presented by a new test in relation to the “reference test” have been frequently used for an in-depth analysis of the false-positive and false-negative results.

12. The interpretation of the agreement analysis is not adequately justified. Although the authors report fair to moderate agreement between PCR–kDNA and other diagnostic methods (κ = 0.38–0.56), this statistic only reflects concordance and should not be used to infer diagnostic superiority or clinical importance. In addition, the statement that only one or two PCR-negative cases were reclassified as positive by IHC, DE, and HE actually suggests a minimal incremental diagnostic yield of these methods over PCR for case confirmation. However, this important observation is not appropriately discussed or critically interpreted. Overall, the manuscript appears to overstate the contribution of IHC, DE, and HE without a balanced evaluation of their true added diagnostic value relative to PCR.

We agree that Kappa coefficient reflects the level of agreement between diagnostic methods and should not be interpreted as evidence of diagnostic superiority or greater clinical value. Accordingly, we have revised the manuscript to clarify, without implying that one method is diagnostically superior to another. To address this point, we revised the Discussion (lines 546-551) to explicitly acknowledge that the main potential value of anti-LACK IHC is not to replace PCR or improve case detection beyond PCR, but to serve as a complementary diagnostic tool in settings where PCR is unavailable or when histopathological evaluation is already being performed as part of the routine investigation of skin biopsies. We also moderated our conclusions throughout the manuscript to avoid overstating the diagnostic contribution of anti-LACK IHC relative to PCR.

13. In addition, the claim of high specificity is not sufficiently critically evaluated, particularly given the absence of cross-reactivity testing with clinically relevant organisms such as Sporothrix braziliensis and Mycobacterium tuberculosis, which is acknowledged but not integrated into the interpretation of diagnostic accuracy.

Thank you for your comment. This is a concern in the development of a new diagnostic test. It is important to explain that cross-reactivity with Sporothrix braziliensis and Mycobacterium tuberculosis was not evaluated in this study, because the non-CL participants were referred to specialized clinical services for confirmatory for other diseases. However, we were able to test cross reactivity only with skin samples from participants of two another studies (DOI: 10.1590/0037-8682-0011-2018 and DOI: 10.1590/0074-02760190324), who were diagnosed with Chagas and Hansen diseases and ethical approval to do IHC technique, according to preview review` comment responses. The evaluation of the potential cross-reactivity with Sporothrix braziliensis would be valuable and should be addressed in future studies designed specifically for this purpose. This limitation can be found in the manuscript (Lines 543-545)

14. The Discussion reads more as a descriptive compilation of studies rather than a critical analysis of the present findings. A clearer structure is needed to separate: (i) what the current study truly demonstrates, (ii) what remains statistically unsupported, and (iii) how these findings realistically impact the diagnostic utility of anti-LACK IHC compared to PCR, DE, and HE.

We have substantially revised and reorganized the Discussion to improve its structure and critical interpretation. We reduced descriptive comparison with previous studies and placed greater emphasis on interpreting the findings of the present study, clearly distinguishing statistically supported results from observations that should be interpreted with caution. We also expanded the discussion of the clinical implications of our findings to provide a more balanced and focused evaluation of the potential role of anti-LACK IHC.

15. Additionally, the conclusion that the assay “successfully detected the main Leishmania species responsible for CL in Brazil” is not fully supported by the presented data, as species-specific diagnostic validation is not clearly demonstrated in the clinical sample set

We highlight that although the anti-LACK IHC had been able to detect the main Leishmania species responsible for CL in Brazil, new study still are necessary to prove its applicability in the CL diagnosis routine in different Brazilian endemic regions

16. What is the significance of conducting both DE and HE experiments separately? In my opinion, performing only one experiment should be sufficient.

Direct examination (DE) and histopathological examination (HE) are distinct diagnostic methods that are routinely used in different clinical and laboratory settings for the diagnosis of cutaneous leishmaniasis. DE is performed on Giemsa-stained tissue imprints obtained immediately after biopsy collection and is primarily used in clinical and public health laboratories for rapid parasitological diagnosis. In contrast, HE is performed on formalin-fixed, paraffin-embedded biopsy specimens processed in pathology laboratories and provides both parasite detection and tissue histopathological evaluation. Because anti-LACK IHC is also performed on formalin-fixed, paraffin-embedded tissue sections, its potential role differs depending on the diagnostic workflow. Comparing anti-LACK IHC separately with DE and HE allows assessment of its added value in relation to each of

Attachment Submitted filename: Response to Review_R2.docx

# Editor report: Decision Letter 2

**DOI:** 10.1371/journal.pone.0339746.r005

**Authors:** Vikash Dubey

8 Jul 2026

Anti- Leishmania-activated C-kinase monoclonal antibody immunohistochemical technique for cutaneous leishmaniasis diagnosis

PONE-D-25-66089R2

Dear Dr. Oliveira,

We’re pleased to inform you that your manuscript has been judged scientifically suitable for publication and will be formally accepted for publication once it meets all outstanding technical requirements.

Within one week, you’ll receive an e-mail detailing the required amendments. When these have been addressed, you’ll receive a formal acceptance letter and your manuscript will be scheduled for publication.

An invoice will be generated when your article is formally accepted. Please note, if your institution has a publishing partnership with PLOS and your article meets the relevant criteria, all or part of your publication costs will be covered. Please make sure your user information is up-to-date by logging into Editorial Manager at Editorial Manager® and clicking the ‘Update My Information' link at the top of the page. For questions related to billing, please contact billing support.

If your institution or institutions have a press office, please notify them about your upcoming paper to help maximize its impact. If they’ll be preparing press materials, please inform our press team as soon as possible -- no later than 48 hours after receiving the formal acceptance. Your manuscript will remain under strict press embargo until 2 pm Eastern Time on the date of publication. For more information, please contact onepress@plos.org.

Kind regards,

Vikash Kumar Dubey

Academic Editor

PLOS One

Additional Editor Comments (optional):

Reviewers' comments:

# Editor report: Acceptance letter

**DOI:** 10.1371/journal.pone.0339746.r006

**Authors:** Vikash Dubey

PONE-D-25-66089R2

PLOS One

Dear Dr. Oliveira,

I'm pleased to inform you that your manuscript has been deemed suitable for publication in PLOS One. Congratulations! Your manuscript is now being handed over to our production team.

At this stage, our production department will prepare your paper for publication. This includes ensuring the following:

* All references, tables, and figures are properly cited

* All relevant supporting information is included in the manuscript submission,

* There are no issues that prevent the paper from being properly typeset

You will receive further instructions from the production team, including instructions on how to review your proof when it is ready. Please keep in mind that we are working through a large volume of accepted articles, so please give us a few days to review your paper and let you know the next and final steps.

Lastly, if your institution or institutions have a press office, please let them know about your upcoming paper now to help maximize its impact. If they'll be preparing press materials, please inform our press team within the next 48 hours. Your manuscript will remain under strict press embargo until 2 pm Eastern Time on the date of publication. For more information, please contact onepress@plos.org.

You will receive an invoice from PLOS for your publication fee after your manuscript has reached the completed accept phase. If you receive an email requesting payment before acceptance or for any other service, this may be a phishing scheme. Learn how to identify phishing emails and protect your accounts at https://explore.plos.org/phishing.

If we can help with anything else, please email us at customercare@plos.org.

Thank you for submitting your work to PLOS ONE and supporting open access.

Kind regards,

PLOS ONE Editorial Office Staff

on behalf of

Professor Vikash Kumar Dubey

Academic Editor

PLOS One
